# Aminoacyl-tRNA synthetase dependent angiogenesis revealed by a bioengineered macrolide inhibitor

**DOI:** 10.1038/srep13160

**Published:** 2015-08-14

**Authors:** Adam C. Mirando, Pengfei Fang, Tamara F. Williams, Linda C. Baldor, Alan K. Howe, Alicia M. Ebert, Barrie Wilkinson, Karen M. Lounsbury, Min Guo, Christopher S. Francklyn

**Affiliations:** 1 Department of Biochemistry, University of Vermont; 2 Department of Cancer Biology, The Scripps Research Institute, Scripps Florida; 3 Department of Pharmacology, University of Vermont; 4 Department of Biology, University of Vermont; 5 Isomerase Therapeutics Ltd, Science Village, Chesterford Research Park, Cambridge CB10 1XL, UK

## Abstract

Aminoacyl-tRNA synthetases (AARSs) catalyze an early step in protein synthesis, but also regulate diverse physiological processes in animal cells. These include angiogenesis, and human threonyl-tRNA synthetase (TARS) represents a potent pro-angiogenic AARS. Angiogenesis stimulation can be blocked by the macrolide antibiotic borrelidin (BN), which exhibits a broad spectrum toxicity that has discouraged deeper investigation. Recently, a less toxic variant (BC194) was identified that potently inhibits angiogenesis. Employing biochemical, cell biological, and biophysical approaches, we demonstrate that the toxicity of BN and its derivatives is linked to its competition with the threonine substrate at the molecular level, which stimulates amino acid starvation and apoptosis. By separating toxicity from the inhibition of angiogenesis, a direct role for TARS in vascular development in the zebrafish could be demonstrated. Bioengineered natural products are thus useful tools in unmasking the cryptic functions of conventional enzymes in the regulation of complex processes in higher metazoans.

Aminoacyl-tRNA synthetases (AARSs) attach amino acids to their corresponding tRNA adaptors with high specificity in an essential reaction of protein synthesis^1^,^2^. In addition, AARSs and AARS-related proteins exhibit diverse alternative activities including RNA splicing, translational regulation, immune system modulation, and angiogenesis^3^,^4^,^5^,^6^,^7^. Recent genetic evidence has served to link AARSs to a variety of human and murine diseases associated with the brain and the nervous system, including Charcot-Marie Tooth disease^8^,^9^, Type III Usher Syndrome^10^, and various encephalopathies^11^,^12^. In several cases, these associations appear to be linked to secondary AARS functions, including several tied to cellular signaling.

One secondary function with significance for human physiology is angiogenesis, where multiple AARSs play a variety of stimulatory and inhibitory modes. For example, human tyrosyl-tRNA (YARS) and tryptophanyl-tRNA (WARS) synthetases are secreted in response to the inflammatory cytokines TNF-α and interferon γ, respectively^6^,^13^,^14^,^15^. Fragments or splice variants of these AARSs exert opposite effects, with the YARS fragment stimulating angiogenesis and WARS inhibiting angiogenesis. While the angiostatic properties of WARS appear to depend on direct interactions with VE-cadherin^16^, a role for AARSs in well-established angiogenic signaling pathways, such as those associated with vascular endothelial growth factor (VEGF), has not been defined. In zebrafish, mutations in the SARS gene encoding seryl-tRNA synthetase are associated with altered vascular development^17^,^18^.

An angiogenic role has recently been identified for the class II threonyl-tRNA synthetase (TARS for eukaryotes; ThrRS for prokaryotic orthologs) that is distinct from those of YARS and WARS. TARS is secreted from endothelial cells in response to TNF-α and VEGF, and potently stimulates angiogenesis in the human umbilical vein endothelial cell (HUVEC) tube formation and chicken chorioallantoic membrane assays^19^. Transwell migration assays also showed that TARS influences angiogenesis by regulating endothelial cell migration. A strong association between TARS expression and advancing stage of ovarian cancer provides evidence that the pro-angiogenic function of TARS in angiogenesis is significant in a pathophysiological context^20^. Currently, the link between canonical aminoacylation function and angiogenesis for TARS is unknown, as is its role, if any, in normal metazoan vascular development.

A class of potent natural products that inhibit the pro-angiogenic properties of TARS represent valuable tools to characterize this function. Borrelidin (BN) (1, Fig. 1) an 18-membered macrolide antibiotic produced in *Streptomyces rocheii*, is a potent antibacterial, antiviral, and antifungal agent^21^,^22^. BN is also a potent anti-malarial^23^,^24^ and inhibits tube formation in a rat aortic angiogenesis model and metastasis in a mouse model of melanoma^25^. The isolation of resistant bacterial strains^26^,^27^ and Chinese hamster ovary cell lines with selective gene amplification^28^ demonstrated that the principal target of borrelidin in bacteria and eukaryotes is threonyl-tRNA synthetase. By contrast, archaeal ThrRSs are highly resistant to BN, a consequence of the significant divergence between these enzymes from those of other kingdoms^29^,^30^. At higher concentrations, BN is known to affect other cellular targets, including the spliceosome associated factor FBP21^31^. Despite its potency, the cytotoxicity of BN to normal epithelial cells has created a significant barrier to any clinical application^32^.

Recently, variants of BN with varying substituents at C17 have been prepared using both bioengineering and semisynthetic approaches^33^,^34^. BC194, which is 100–1000 fold less toxic to endothelial cells than BN, notably retains the ability to block angiogenesis^19^,^34^. The mechanistic basis of this difference in toxicity remains to be determined. Increased understanding of the molecular basis for the difference between BN and BC194 may allow application of these compounds in a clinical setting, such as in the therapeutic inhibition of angiogenesis. Here, we demonstrate that the smaller C17 ring of BC194 weakens its interactions with essential TARS catalytic residues, thereby reducing its ability to compete with threonine for binding. Direct comparison of the effects of BN and BC194 on cells reveals that the toxicity differences originate from the varying ability of the macrolides to elicit the amino acid starvation response. At the same time, both compounds exhibit similar potency in blocking angiogenesis at sub-toxic concentrations. These observations argue against apoptosis as the sole mechanism for BN’s anti-angiogenic activities^35^. Finally, we compare the effects of BN derivatives on vascular development in the zebrafish, and provide the first direct evidence for the role of TARS in angiogenesis *in vivo.*

## Results

### BC194 displays weakened interactions with the amino acid binding pocket in the TARS active site relative to BN

**Figure 1:**
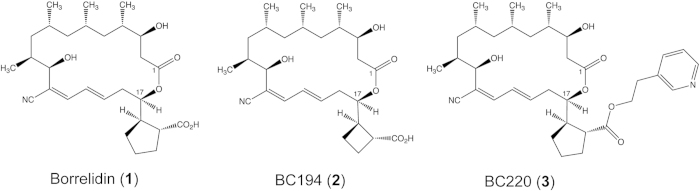
Structures of macrolides used in this study. Structures of borrelidin BN (1), BC194 (2), and BC220 (3).

**Figure 2:**
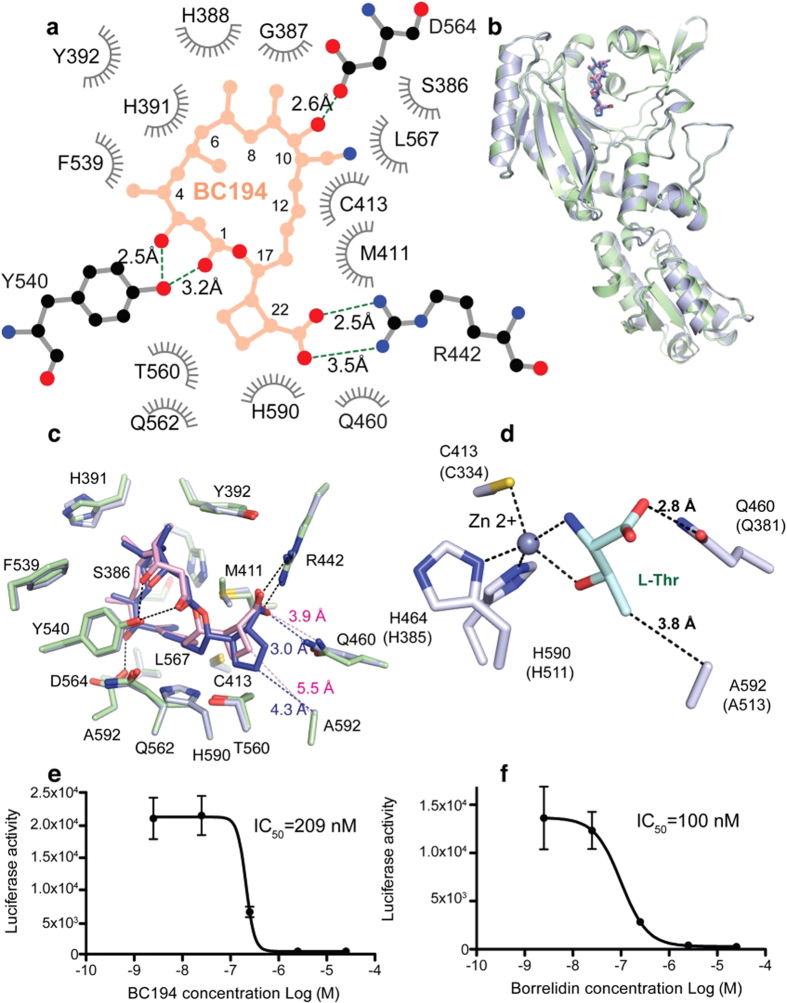
Structure of TARS-BC194 complex. (**a**) Two-dimensional scheme of TARS-BC194 interactions. H-bonding residues are shown as sticks. Hydrophobic interacting residues are shown in grey. (**b**) Structure superimposition of TARS-BC194 (green) and TARS-BN (grey) complexes. The protein is shown in ribbon cartoon representation, and the bound BC194 and BN are shown as pink and blue sticks, respectively. (**c**) Close up view of BC194 (green) and BN (grey) binding site residues. The five shared H-bonds are shown as black dash lines. The 2 BN-specific interactions are shown as blue dash lines, while the corresponding distances in BC194 structure are indicated in pink. (**d**) Close up view of threonine binding interactions. Interactions are shown as dashed lines. (**e,f**) The effects of BC194 (**e**) and BN (**f**) treatment on a cell-free translation system. Rabbit reticulocyte lysate (RRL) was incubated with 0.02 mg/ml luciferase mRNA and translation of luciferase enzyme was quantified in a luminescence assay. Serial diluted BC194 and borrelidin (2.5 nM - 25 μM) was added to inhibit the translation of luciferase mRNA; mean ± SEM, n = 3.

The molecular cloning of the BN biosynthetic operon from *Streptomyces parvulus* Tu4055^36^ permitted novel variants of BN to be produced through biosynthetic engineering^33^,^34^. In BC194, a cyclobutane ring replaces the pendant C17 cyclopentane ring (2, Fig. 1). Relative to other less effective variants, BC194 retained potent inhibition of angiogenesis while possessing substantially reduced toxicity towards endothelial cells^34^. As a first step towards understanding the molecular basis of these effects, we co-crystallized BC194 with a fragment of human TARS comprising the catalytic and anticodon binding domains, and solved the structure to a resolution of 2.8 Å (Table S1). The structures of BN and BC194 differ at position C17, with BN containing a pendant cyclopentanecarboxylic acid ring, and BC194 a cyclobutanecarboxylic acid ring (Fig. 1). BC194 binding to the TARS active site is stabilized by numerous Van der Waals interactions and five distinct enzyme-compound hydrogen bonds (Fig. 2a). In addition, BC194 induces a conformation of TARS close to that of BN – TARS complex, with an r.m.s.d. of 0.62 Å between superimposed BC194 and BN – TARS complex structures (for all 402 Ca’s in TARS) (Fig. 2b)^37^. In a global structural sense, BN and BC194 act to stabilize the same conformational state for TARS, with potential consequences for secondary functions (*vide infra*).

Interactions between TARS and the C4 to C14 moiety of the macrolide structure are conserved in the BN and BC194-TARS complexes, including hydrophobic contacts to the macrolide ring made by L567, S386, H388, Y540, D564, Q562, H590, Y392, H391, and F539 (Fig. 2c) (Table S2). In complexes with both compounds, residues T560, R442, M411, C413, Q460 and A592 comprise a binding pocket outside the macrolide ring, and interact with the C17 cyclobutanecarboxylic acid ring. The high structural similarity between the BN and BC194-TARS complexes rationalizes previous findings that homologous substitutions in the *E. coli* and human enzymes (L489W and L567V, respectively) give rise to BN and BC194 resistant versions of the enzyme^19^,^29^.

The key structural difference that differentiates how BN and BC194 interact with TARS is seen in the contacts made to the respective pendant rings. In the BC194 complex, the absence of a methylene group in the smaller cyclobutane ring lengthens the contact between the C17 carboxylic-oxygen atom and the 5-amide nitrogen atom of Q460 by 0.9 Å. A strong hydrogen bond normally found in the BN complex is eliminated, and the hydrophobic interaction between the cyclopentane ring and A592 is also weakened (Fig. 2c). Based on the prokaryotic ThrRS complexes, Q460 and A592 are both predicted to make key H-bond and hydrophobic interactions, respectively, with L-Thr^38^ (Fig. 2d). The loss of the interactions with these two residues suggests that BC194 may compete less effectively against threonine for binding than the parent compound BN. The ability of threonine to rescue the inhibition of translation by both compounds was compared in a cell free protein synthesis assay, and this indicated that the IC_50_ for BC194 is increased two-fold relative to BN (Fig. 2e,f). In the context of a cell free protein synthesis system, the weaker competition by BC194 for amino acid binding site residues could thus be shown to have direct consequences for inhibition.

### BN mediated amino acid starvation elicits cell cycle arrest

BN and BC194 differ in their toxicity to endothelial cells, an effect not previously characterized at the biochemical level^34^. While BC194 retains the potent (3.7 nM) inhibition of TARS that is characteristic of BN, a significant global shutdown of protein synthesis was not observed^19^. In order to better understand the physiological consequences of BN (1) and BC194 (2), we compared their effects on HUVEC cell-cycle progression using flow cytometry (Fig. S1a,b). Treatment with 10 nM BN arrested cells at the G_0_ boundary, delaying progression into G2/M up to 24 h. In contrast, cells treated with 10 nM BC194 did not significantly deviate from serum treated controls, and entered the G2/M phase 16 h following serum exposure (Fig. S1b). The effects of BN and BC194 on HUVEC proliferation were subsequently investigated using an alamarBlue® based assay (Fig. S1c). Reductions in cell proliferation were observed at concentrations as low as 10 nM for BN, whereas a 10-fold higher concentration of BC194 was required for a comparable decrease in cell proliferation. Thus, a major component of BN’s toxicity arises from its ability to block cell cycle progression.

**Figure 3:**
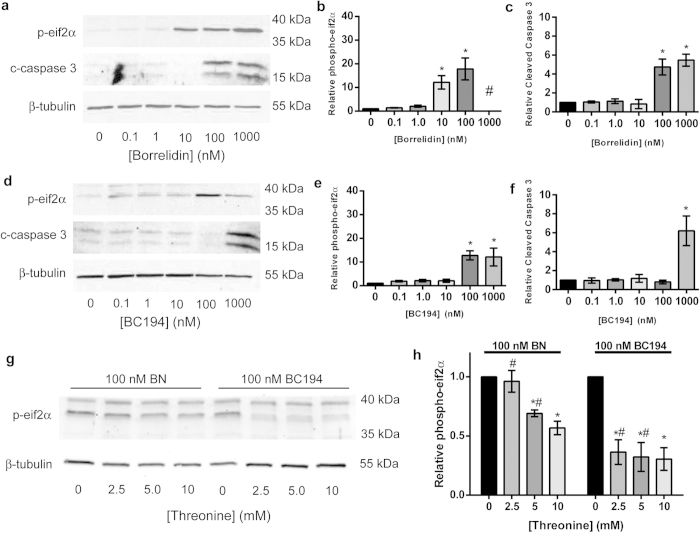
The cytotoxicity of borrelidin is linked to the induction of the amino acid starvation response. (**a–f**) HUVEC cells grown in full serum media were exposed to the indicated concentrations of BN (**a–c**) or BC194 (**d–f**) and standardized to 0.05% DMSO. Cropped images from western blots of cell extracts were analyzed using antibodies recognizing phospho-eIF2α and cleaved-caspase 3 with β-tubulin as a loading control. Full images of blots can be found in Supplementary Figures S2a and S2b. Quantification of phospho-eIF2α and cleaved-caspase 3 for BN (**b,c**) and BC194 (**e,f**) relative to β-tubulin; mean ± SEM, n ≥ 3, *p < 0.05 relative to 0 nM (one-way ANOVA, Tukey Test). The apparent drop in the levels of phospho-eif2α as BC194 increased to 1000 nM and the exclusion of 1000 nM BN-treated data (designated by #) are due to the fact that endothelial cells exposed to high macrolide concentrations were visually apoptotic, making estimations of total protein loaded difficult. As such, we believe that the variability observed in these data at higher concentrations was more related to the severe status of the cells rather than a change in the amino acid starvation response. (**g,h**) Western blot (**g**) quantification of eif2α amounts relative to β-tubulin for both BN- and BC194-treated cells (100 nM) in the presence of various threonine concentrations (0–10 mM); mean ± SEM, n ≥ 3, *p < 0.05 relative to 0 mM threonine (one-way ANOVA, Tukey Test), #p < 0.01 between compounds at same threonine concentration (two-way ANOVA, Sidak Test). Full images of blots can be found in Supplementary Figure S2c. See also Supplementary Figure S1 for cell cycle, proliferation effects, and Supplementary Figure S3 for BC220 data.

We hypothesized that the ability of the two compounds to induce cell cycle arrest is linked to how effectively each macrolide induces amino acid starvation. Accumulation of uncharged-tRNA within the cell arising from the inhibition of aminoacylation increases uncharged tRNA levels, which activates the EIFAK4 (GCN2) translational control kinase^39^,^40^. Activation of EIFAK4 increases the phosphorylation of the downstream translational initiation factor eIF2α on Ser51, triggering additional ER stress and unfolded protein response pathways^39^. BN is known to de-repress expression of amino acid biosynthetic genes in yeast, a signature of GCN2 activity^41^. Particularly when coupled to the unfolded protein response, amino acid starvation leads to cell cycle arrest and, in severe cases, the initiation of apoptosis^42^,^43^,^44^. To confirm that the anti-proliferative effects of BN are linked to AAS pathways in animal cells, cultured HUVECs were exposed to increasing concentrations of BN or BC194, and then lysates from these cultures were probed for the starvation and apoptosis markers phospo-eIF2α and cleaved-caspase 3, respectively (Fig. 3a,d, S2a,b). The phosphorylation of eIF2α was induced at concentrations as low as 10 nM BN (Fig. 3b) but at least 10-fold higher concentrations of BC194 were required to generate an equivalent response (Fig. 3e). Likewise, the appearance of cleaved-caspase 3 at 100 nM BN (Fig. 3c) compared to 1000 nM for BC194 (Fig. 3f) demonstrates the greater potency of BN relative to BC194 in inducing apoptosis. BN therefore blocks progression through the cell cycle and induces amino acid starvation and apoptosis at 10-fold lower concentrations than were required for BC194.

As described above, the binding sites for both BN and BC194 overlap with those of all three substrates, including threonine. We hypothesized that the induction of amino acid starvation by anti-AARS inhibitors is linked to their extent of aminoacylation inhibition, and the sensitivity of that inhibition to substrate competition. We therefore examined the efficiency of induction of amino acid starvation by BN and BC194 in the presence of increasing threonine concentrations. Addition of threonine over a concentration range of 2.5–10 mM to HUVECs treated with 100 nM BC194 decreased the phosphorylation of eIF2α to untreated levels (Fig. 3g,h, S2c). By contrast, addition of this same range of threonine concentrations to HUVECs treated with 100 nM BN failed to decrease the levels of eIF2α phosphorylation to the same extent as with BC194. This combined structural and biochemical analysis suggests that the reduced ability of BC194 to inhibit protein synthesis compromises its induction of amino acid starvation in HUVECs, thereby alleviating the toxic growth and proliferation effects seen with BC194.

As an additional control to demonstrate that BN induced amino acid starvation response is directly linked to TARS inhibition, HUVECs were incubated with a third compound, BC220 (3), which is structurally distinguished from BN by a 3-ethyl piperidine group that is esterified to the cyclopentane carboxylic acid moiety (Fig. 1). The K_i_ for inhibition of TARS aminoacylation by BC220 is greater than 10 μM, which is nearly 1000 times greater than that of BN or BC194^45^. HUVECs exposed to BC220 showed no induction of eIF2α phosphorylation or cleavage of caspase 3 (Fig. S3a–d), indicating that the lack of physiological response is highly correlated with its failure to inhibit TARS. In summary, the cytotoxicity of BN is linked to the activation of the amino acid starvation response and apoptosis, which originates from inhibition of TARS.

### BN and BC194 exhibit comparable inhibition of angiogenesis at sub-toxic levels

**Figure 4:**
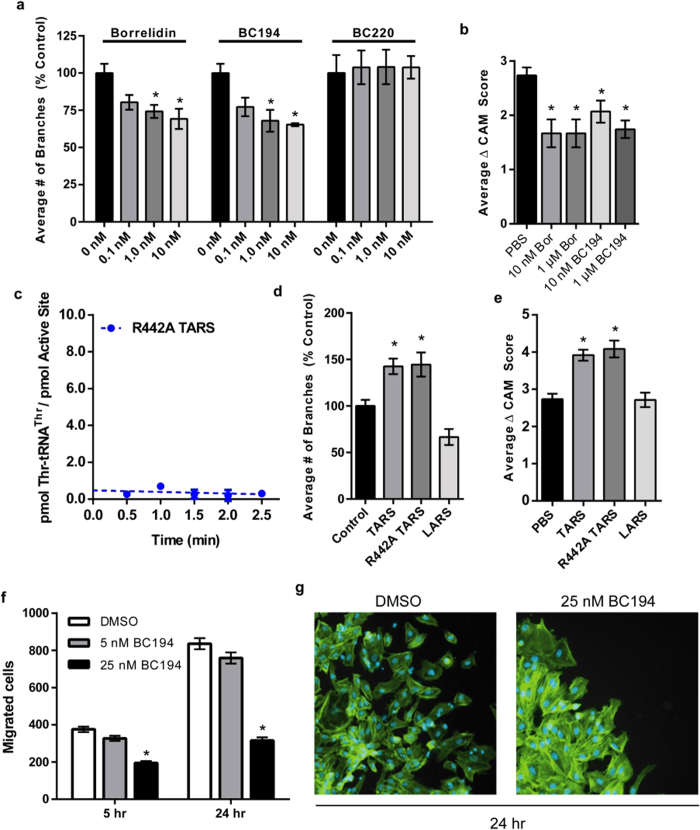
BN and BC194 exhibit comparable inhibition of angiogenesis at sub-toxic levels. (**a**) Quantification of HUVEC branching at varying concentrations of BN, BC194, and BC220. HUVECs were plated on Matrigel in full EGM-2 (2% FBS) media and exposed to the indicated concentrations compounds. Cells were fixed after 4–8 h and stained with Oregon Green 488 Phalloidin. Numbers represent percentage of control (0 nM) samples, mean ± SEM, n = 3 *p < 0.05 relative to 0 nM (one-way ANOVA, Tukey Test for each compounds). No significant differences were observed between 0.1, 1, and 10 nM samples within the same treatment or between BN and BC194-treated samples at the same concentrations (t-test for each treatment pair, not indicated in figure). (**b**) The effects of BN and BC194 on *in vivo* angiogenesis in a CAM assay. Fertilized chicken embryos were cultured *ex-ova* for 10 days after which compounds at the indicated concentrations were applied to gelform sponges on the CAM. Images were taken daily over 72 h and quantified as the change in vascularity score over this entire period; mean ± SEM, n ≥ 12, *p < 0.05 relative to PBS (one-way ANOVA, Tukey Test). (**c**) Aminoacylation activity data for R442A TARS. Numbers represent the formation of Thr-tRNA^Thr^ per active site (pmol/pmol) over time; mean ± SEM, n = 3. (**d,e**) Quantification of HUVEC branching (**d**) change in CAM vascularity (**e**) in response to exogenous wildtype TARS, the catalytically R442A TARS, and LARS; mean ± SEM, n = 4, *p < 0.05 (Tube Formation), n ≥ 12, *p < 0.01 (CAM). (**f**) Quantification of migrated cells exposed to 5 or 25 nM BC194 after 5 and 24 h in the donut assay; mean ± SEM, n ≥ 6, *p < 0.01 relative to DMSO of same time point (one-way ANOVA, Tukey Test). (**g**) Image of cells at the leading edge of the migratory boundary after 24 h exposure to DMSO (left) or 25 nM BC194 (right). See also Supplementary Figure S4, S5, and S6 for representative images.

Previous studies indicate that BN and BC194 are both potent inhibitors of angiogenesis^25^,^34^,^46^. We wondered whether the anti-angiogenic effect is a direct consequence of the induction of the amino acid starvation response in endothelial cells, leading to their self-destruction through apoptosis, or is instead a reflection of another physiological effect. The ability of all three macrolides to inhibit HUVEC branching at sub-toxic concentrations was therefore tested in the endothelial tube formation assay, a cellular model of angiogenesis (Fig. 4a, S4a,c). At concentrations as low as 1 nM, significant decreases in branch formation were observed for both BN (1) and BC194 (2) relative to uninhibited conditions. Notably, these concentrations are 10- and 100- fold below the lowest BN and BC194 concentrations required to induce eIF2α phosphorylation (Fig. 3). Exposure to BC220 (3) at these same concentrations had no effect on tube formation (Fig. 4a). BN and BC194 thus exhibit comparable potencies in inhibiting angiogenesis, despite the differences between their interactions with the TARS active site.

We extended these results by using the chorioallantoic membrane (CAM) assay, which monitors basal vessel formation in the fertilized chicken egg and thus more closely approximates *in vivo* angiogenesis, relative to the simple HUVEC tube formation assay. As shown in Fig. 4b, recurring administration of BN or BC194 over the range of 10 nM to 1 μM to CAMs for a 72 h period inhibited basal vessel formation relative to the PBS control. Notably, between 10 nM – 1 μM, BN and BC194 exhibited comparable potencies, consistent with the HUVEC tube formation results (Fig. 4a). As controls, equivalent concentrations of DMSO (0.01%), did not differ from the PBS treatment (Fig. S5a). At concentrations of 100 μM for either macrolide, necrosis was clearly evident in the BN treated CAMs, but not those treated with BC194 (Fig. S5b). BC194 therefore appears to retain all of the potency of BN in the inhibition of angiogenesis, while exhibiting substantially less cytotoxicity. Accordingly, the inhibition of angiogenesis by BN and BC194 appears not to be specifically dependent on the activation of the AAS, and its subsequent induction of apoptosis.

These results prompted us to consider whether the observed angiogenic properties of TARS are distinct from the well-characterized aminoacylation function. We thus prepared a mutant version of TARS that is predicted to lack aminoacylation function, while still retaining any putative secondary functions. Arginine 442 is highly conserved in all Class II ARSs and is required to stabilize the adenylate transition state in the first step of aminoacylation^47^. As predicted, substitution of Arginine 442 with alanine in R442A TARS completely eliminated the aminoacylation activity of the enzyme (Fig. 4c). By contrast, the addition of R442A TARS significantly increased HUVEC branch formation (Fig. 4d, S4b) and basal vessel formation (Fig. 4e) in tube-formation and CAM assays, respectively. Notably, a leucyl-tRNA synthetase (LARS) non-specific ARS control did not significantly affect tube formation or CAM vascular development (Fig. 4e). Together, these data establish that TARS angiogenic function is independent of its classical role in providing aminoacylated tRNA for protein synthesis.

In a previous study, exposure of HUVEC cultures to exogenous TARS in transwell migration assays increased cell migration, a component of angiogenesis^19^. Other earlier work showed that borrelidin inhibits tumor cell migration^48^. We therefore tested the effect of BC194 on cell migration using a donut migration assay^49^. In this assay, endothelial cells are confined by a cloning ring on a fibronectin-coated dish, and then cells that migrate after ring removal are quantified by image subtraction (Fig. S6a,b). The number of migratory cells was significantly lower in cultures treated with 5 nM BC194 for 5 h and 25 nM BC194 for either 5 or 24 h, relative to untreated cells (Fig. 4f). Interestingly, close inspection of the cells at the leading edge of each sample reveals a marked increase in cell-cell contacts in samples exposed to 25 nM BC194 relative to DMSO (Fig. 4g). These results support prior observations that an important component of TARS angiogenic function is the stimulation of endothelial cell migration^19^.

### BC194 induces specific ectopic blood vessel formation in zebrafish without the developmental toxicity associated with borrelidin

Inspection of the surface vessels proximal to the BN or BC194 treated sponges in the CAM assay revealed instances of directional changes during vessel migration (Fig. S7a). These directional changes often came in sets of three, potentially corresponding to three cycles of compound addition and clearance over the 72-hour period. Such effects led us to propose that TARS promotes endothelial cell migration in a normal developmental context, potentially as part of directional growth and patterning. Inhibition of this function by BN and its derivatives causes an irregular vessel architecture to result.

We tested this hypothesis by exposing transgenic zebrafish embryos derived from the *Tg(flk: eGFP)* or *Tg(flk: dsRed)* lines to a range of concentrations of BN, BC194, and BC220 13–15 h post fertilization. After 24 hours of compound exposure (hours post exposure, hpe), significant changes in gross morphology were observed in embryos exposed to BN (1) (left column) but not BC194 (2) (middle column) or BC220 (3) (right column) (Fig. S7b). The toxicity of these treatments was assessed by measurements of body length (Fig. S7c) and heart rate (Fig. S7d). Significant intra-group decreases were observed for fish treated with either BN or BC194, but not BC220. However, inter-group analyses revealed significant reductions in heart rate and body length in BN-treated relative to BC194-treated embryos starting at concentrations of 2.5 μM and 1 μM respectively (not denoted in figure), indicating that BN had a more dramatic effect on toxicity than BC194.

**Figure 5:**
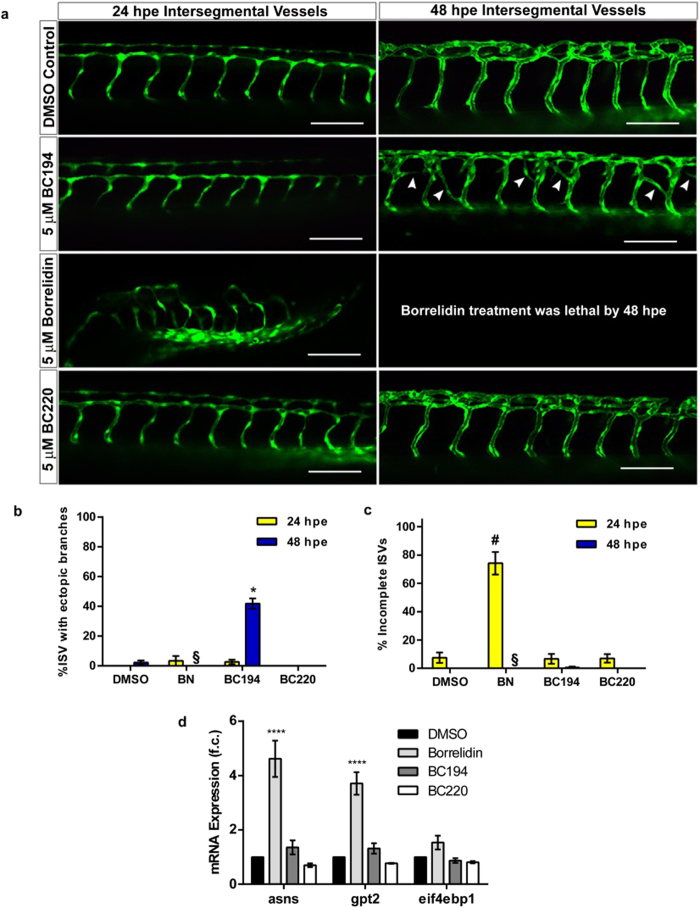
Exposure to BN and BC194 results in vascular defects and mis-patterning. (**a**) Representative confocal images (20x magnification) of zebrafish ISVs at 24 and 48 hpe. Zebrafish embryos were incubated in egg water at 25 °C until 8–12 somites of age. The embryos were then manually dechorionated and incubated in egg water containing the indicated compounds at 28.5 °C until the images were taken. Calibration bar represents 100 μm. (**b,c**) Quantification of ectopic branching (**b**) and aberrant patterning (**c**) of ISVs. Embryos were incubated with the indicated compounds (5 μM) or DMSO until images were taken by fluorescent microscopy at 24 and 48 hpe. Aberrant structures were then counted within a region encompassing 5 ISVs anterior and posterior from the end of the yolk extension; mean ± SEM, n ≥ 8, ^#^*p < 0.0001 relative to DMSO at 24 and 48 hpe respectively (one-way ANOVA, Tukey Test). ^‡^BN-treated fish rarely survived to 48 hpe. (**d**) RT-qPCR values for the expression amino acid starvation response genes *asns*, *gpt2*, and *eif4ebp1*. Dechorionated embryos (15 somites) were incubated in egg water containing the indicated compounds for 24 hours. Total RNA was extracted by trizol/chloroform and the expression levels relative to *actb were* determined using the ΔΔCT method; mean ± SEM, n = 3, ****p < 0.0001 relative to DMSO (two-way ANOVA, Tukey Test). See Supplementary Figure S7 for physiological and toxicity information.

The angiogenesis-related effects of these compounds were assessed by exposing the embryos to 5 μM concentrations of the various macrolides, followed by screening using fluorescence microscopy for development defects in trunk inter-segmental vessels (ISVs) (Fig. 5a). Significant patterning defects were observed in response to either BN or BC194 treatment. Ectopic branches readily formed in the ISVs of embryos exposed to 5 μM BC194 for 48 hpe, but not in those of BN-treated embryos (Fig. 5b). By contrast, the ISVs in BN-exposed fish were often truncated or incomplete at both 24 and 48 hpe (Fig. 5c). Significantly, these effects were not observed when embryos were treated with the control BC220 macrolide or the DMSO carrier (Fig. 5a–c). One explanation for the diminished toxicity of BC194 relative to BN is that, as seen with in animal cell culture, BC194 has a reduced ability to induce amino acid starvation in the context of a whole animal model. To test this hypothesis, quantitative real time PCR was performed on total RNA prepared from embryos treated with equivalent (5 μM) concentrations of BN, BC194, and BC220, and the DMSO vehicle. The ATF4 target genes *asns*, *gpt2*, and *eif4ebp1* served as AAS markers^43^. Consistent with our model, BN caused a significant increase in the expression of the markers *asns* and *gpt2*, while BC194 did not (Fig. 5d). Thus, BC194’s induction of ectopic ISV formation in the zebrafish is not dependent on the induction of the amino acid starvation response.

In addition to faulty vessel patterning, a reduction in vessel lumen formation was also observed in embryos treated with either 5 μM BN or BC194 for 24 hpe and remained partially incomplete even at 48 hpe. This observation was confirmed by live videos of blood flow in trunk vessels of 48 hpe embryos treated with BC194 or DMSO. No restriction of blood flow was observed in ISVs of DMSO-treated fish (Video S1) consistent with fluorescent images showing well-defined lumens (Fig. S8a). In contrast, several ISVs in embryos treated with 5 μM BC194 were non-functional in terms of blood flow (Video S2) and appeared to correspond to vessels associated with ectopic branches and poorly-defined lumens (Fig. S8b).

### A direct role for TARS in vascular development in the zebrafish

The results of the prior experiments argue that the faulty patterning and reduction in vessel lumen formation seen with BC194 in the zebrafish may originate from a TARS function distinct from aminoacylation. To obtain further insights into the role of TARS in angiogenesis, we investigated the effects of BN (1), BC194 (2), and BC220 (3) on the expression of *tars* and *vegfaa* in zebrafish embryos. Additionally, proper vessel patterning requires a limiting of the number of endothelial cells that are permitted to lead the migration of new vessels. These restrictions are primarily mediated through Delta-Notch signaling between the lead cells, known as tip cells, and the adjacent stalk cells^50^. Since the dysregulation of this system has been shown to cause excessive vessel branching we also measured the expression of the Notch-controlled genes *ephrinb2a* and *heyL*^51^,^52^. Quantitative real time PCR (RT-qPCR) of total embryo RNA revealed significant increases in the expression of both *tars* and *vegfaa* (Fig. S9a,b) mRNA after exposure for 24 hours with BN or 48 hours with BC194. No changes in gene expression were observed for embryos treated with either DMSO or BC220, consistent with our TARS-dependent nature of our current model for BN and BC194 action. Interestingly, the *notch* reporter genes showed no change over any of the tested conditions (Fig. S9c,d).

**Figure 6:**
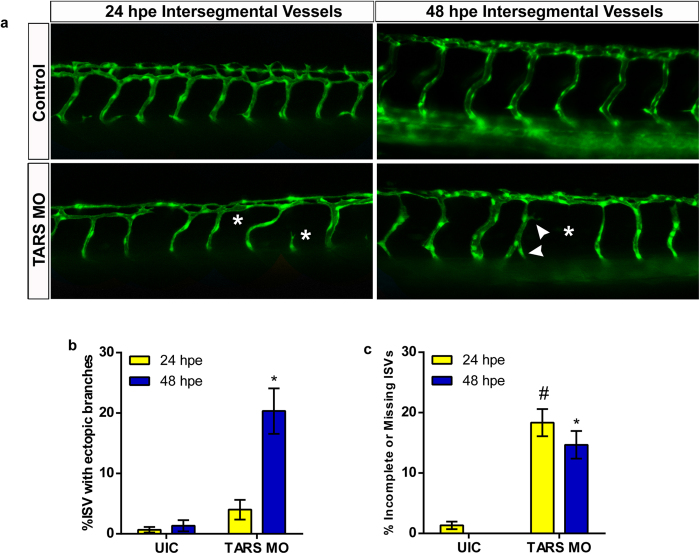
TARS is involved in vascular development in the zebrafish. (**a**) Representative confocal images (20× magnification) of control and TARS morphant zebrafish ISVs at 24 and 48 hpe. Embryos were injected with a TARS morpholino (1.5 μM) at the one to four cell stage. Fish were manually dechorionated at 24 h after fertilization and imaged at 24 and 48 hpe. Arrows and asterisks denote the location of ectopic branches and missing/incomplete vessels respectively. (**b**,**c**) Quantification of ectopic branching (**b**) and missing/incomplete ISVs (**c**) from control and morphant zebrafish within a region of encompassing five ISVs anterior and posterior to the yolk extension; mean ± SEM, n ≥ 29, ^#^*p < 0.0001 relative to uninjected controls at 24 and 48 hpe respectively (one-way ANOVA, Tukey Test). See Supplementary Figure S10 for morpholino validation and morphological effects.

Previous work demonstrated that point mutations in TARS can block the anti-angiogenic effects of BC194, thereby indicating that TARS is the specific target of BC194^19^. To confirm that the vessel patterning effects of BN and BC194 in zebrafish are a direct consequence of action on TARS, we reduced the levels of functional TARS by some 62% using a splice altering antisense morpholino oligonucleotide (MO) (Fig. S10a–c). Consistent with our hypothesis, morphant fish developed with significantly more ectopic branches and incomplete vessels than uninjected controls (Fig. 6a–c). Phenotypically, these effects on the vasculature were intermediate with respect to the previously shown (Fig. 5a) effects of BN and BC194. Notably, the TARS morphant fish did not exhibit the severe body morphology associated with BN toxicity (Fig. S10d). In summary, these results demonstrate that vascular development in the zebrafish is highly sensitive to alterations in the activity of TARS, induced either as a result of chemical inhibition or altered gene expression. We conclude that TARS has a direct role in vertebrate vascular development.

## Discussion

Natural product and synthetic inhibitors have been identified for many AARSs, and readily exploited to generate anti-microbial infectives^53^,^54^, immunosuppressive agents^43^,^55^, and potential modulators of cancer metastasis^56^. However, many natural compounds that target AARSs have a very narrow therapeutic index, dramatically limiting their clinical usefulness. The reported toxicities may reflect the acute sensitivity of animal cells to the inhibition of protein synthesis, and subsequent cell cycle arrest and apoptosis^42^,^43^.

Borrelidin’s (BN’s) pleiotropic effects on mammalian cells have been appreciated for decades, but the molecular basis of its action has been poorly understood^34^. Here, a chemical and structural biology approach was used to compare the effects of BN and two different derivatives, and gain insights into their ability to inhibit angiogenesis. Significantly, the cytotoxicity of BN was found to originate from its induction of amino acid starvation, which leads to cell cycle arrest and apoptosis. The ability of BN to induce amino acid starvation in yeast has been reported earlier^41^. In animal cells, amino acid starvation induces the expression of the CCAAT/enhancer binding protein homology binding protein (CHOP), a transcription factor that stimulates apoptosis after prolonged nutritional deprivation^57^. It is thus not surprising that BN potently induces apoptosis in acute lymphoblastic leukemia cells^58^. Our studies therefore support a strong linkage between the ability of a given aminoacyl-tRNA synthetase inhibitor to induce the amino acid starvation response, and its potency with respect to apoptosis and cell killing.

The actions of BN and BC194 can be ablated by single residue substitutions in the *E. coli* ThrRS^29^, and human TARS active sites^19^, respectively, arguing against the previous suggestion that BN binding to other secondary targets is physiologically relevant to the inhibition of angiogenesis^46^. For both BN and BC194, occlusion of the binding sites for the three canonical aminoacylation substrates (Thr, ATP and tRNA), as well occupancy of a fourth subsite distal to the substrates is integral to their mechanism of inhibition^30^. This diverse set of interactions rationalizes the previously reported slow tight binding inhibition kinetics^29^, as well as observations that high threonine levels *in vivo* diminish inhibition.

The major structural difference between the BN- and BC194-TARS (Fig. 2d) complexes is that the latter exhibits significantly weaker interactions with the threonine binding pocket^38^,^59^. Threonine was more efficient at blocking amino acid starvation in BC194-treated cells relative to those treated with BN (Fig. 3g,h), suggesting that BC194’s increased susceptibility to threonine competition rationalizes toxicity differences between the two compounds. Additionally, a further contributor to BC194’s reduced toxicity might be its decreased stability, owing to increased strain of the cyclobutane versus cyclopropane pendant rings. While currently available animal data have not provided direct support for that hypothesis^45^, additional pharmacokinetic studies would be needed to provide a definitive answer. BC194’s potency relative to BN as an inhibitor of angiogenesis, however, was undiminished. Thus, while competition between the BN macrolide inhibitors and threonine accounts for potent stimulation of the amino acid starvation response, and the toxicity of the inhibitors, angiogenesis inhibition is apparently less sensitive to such competitive effects.

Additional evidence for the separation of pro-angiogenic effects from aminoacylation was provided by experiments using aminoacylation defective R442A TARS. R442A TARS retained the ability to stimulate angiogenesis with a potency that was essentially unchanged with respect to wild type TARS (Fig. 4c). One hypothesis is that BN and BC194 inhibit angiogenesis by virtue of their stabilization of a TARS conformation (Fig. 2b) that is distinct from unliganded and substrate bound states^30^. Such a model would be consistent with the observation that the stimulation of angiogenesis depends on the structural integrity of the catalytic domain, and its potential to associate with additional protein partners. It also predicts that TARS amino acid substitutions that stabilize the BN-dependent open conformation would exhibit reduced angiogenesis function, which is currently under examination (A.M, M.G., and C.F, unpublished observations).

An additional major finding of this study is that the reduction of TARS activity via BC194 administration and decreases in steady state TARS levels via antisense morpholino both produced similar phenotypes, including incomplete vessel development, increased ectopic branching, and decreased vessel lumens (Figs 5 and 6, Movies S1, S2). An attractive hypothesis is that the inhibition and/or reduction of TARS elicits anti-angiogenic effects by the reduction of TARS-dependent vessel guidance signals that are required to build a regular vascular architecture during development. If correct, this would represent one of the first examples where an AARS is required as a pro-angiogenic factor during the vascular development of a metazoan. Treatment with BC194 also led to increased expression of TARS (Fig. S7a), also seen in the response of CHO cells to persistent exposure to BN^28^. In view of the relatively high macrolide concentrations employed (i.e. several orders of magnitude over the apparent K_i_), the increased TARS protein levels resulting from BC194 administration might effectively be masked by the stoichiometric formation of TARS: BC194 complexes, which are characterized by very slow off rates^29^. Alternatively, the disruption in TARS levels resulting from BC194 treatment could alter a TARS dependent concentration development gradient that is necessary for proper guidance and patterning of the developing vasculature^19^,^60^,^61^.

Along these lines, endothelial cells treated with BC194 showed a higher degree of cell-cell contacts relative to controls, even at the leading edge of a migratory boundary (Fig. 4d). While these observations provide an apparent correlation between inhibition of endothelial cell migration and increased cell-cell contacts, the direct or indirect basis of TARS involvement remains to be determined. Weakened cell-cell contacts have been proposed to be an important component of angiogenesis^62^. Inhibition of tyrosine phosphorylation on VE-cadherin, which alters cell-cell interactions, decreases VEGF-mediated migration and angiogenesis in cell culture^63^. Furthermore, certain models of lumenogenesis involve the strictly regulated disruption of contacts between endothelial cells of the developing vessel^64^. BN and related macrolide compounds may act to disrupt TARS-mediated regulation of cell-adhesion proteins, thereby preventing both migration and proper vascular development^48^. Specific inhibitory compounds and TARS variants that ablate its angiogenic function should prove to be valuable tools to critically test this hypothesis.

In addition to TARS, other aminoacyl-tRNA synthetases exhibit angiogenesis related functions that are distinct from aminoacylation^65^. These include the glutamyl prolyl-tRNA synthetase (EPRS), which is an integral component of the GAIT complexes that regulates VEGF translation^4^,^66^, and tryptophanyl-tRNA synthetase (WARS), which apparently binds VE-cadherin to inhibit angiogenesis^67^. Of greater interest is the closely related seryl-tRNA synthetase (SARS), where a loss-of-function substitution in SARS causes a derepression in *vegf* transcriptional control, increasing intersegmental vessels^17^,^18^. The finding of angiogenesis related functions among numerous AARSs, and the potent modulation of those functions by natural products, indicates the potential value of AARSs as general therapeutic targets for diseases where blocking vascular development may be desirable, such as cancer.

## Methods

### Cell Culture and Reagents

Human umbilical endothelial cells (HUVECs) were maintained in Clonetics EGM®-2 complete media (Lonza) and used between passages 5 to 8. The anti-TARS morpholino was purchased from GeneTools. BN and BC194 were obtained from Isomerase Therapeutics Ltd (Cambridge, UK)). BC220 was a gift from Dr. Llouis Ribas de Pouplana. Owing to their poor solubility in aqueous solutions all three macrolide compounds were stored at high concentrations in DMSO and diluted in aqueous solutions for experiments. Depending on the individual experiment, final concentrations of DMSO ranged from 0.0001% to 0.025% for BN and BC194 and 0.05% to 0.2% for BC220 because of its more dilute stock concentration.

### Flow Cytometry

HUVECs were plated onto 10 cm dishes and serum starved in 0.5% FBS EGM®-2 media for 24 h. Cells were pre-treated with 10 nM BN or BC194 for 6 h. To assess the serum-induced cell cycle entry, full serum media (EGM®-2 complete media) was added at staggered times, such that all samples were collected simultaneously for the 8 h, 16 h and 24 h time points. Cells were trypsinized, washed and fixed in 80% ethanol, washed in 2% BSA and then incubated with a solution containing 10 μg/ml propidium iodide (PI) and 250 μg/ml RNAse A for 16 h as in^68^. Analysis of the PI signal (620 nm) was performed on an EPICS XL four-color analytical flow cytometer coupled with EXPO32 ADC software.

### Endothelial Cell Proliferation Assay

The MTT-based alamarBlue® (Invitrogen) reagent was used to assess cell proliferation^69^. HUVECs were seeded in a 96-well dish (1 × 10^3^ cells/well) and grown for 48 h in EGM®-2 media. Cells were incubated with BN or BC194 (0.1–1000 nM) as indicated; media alone served as the control. After 48 h in culture, 10 μl/well premixed alamarBlue® was added and after 3 h at 37 °C the amount of reduced alamarBlue® was quantified by fluorescence (excitation at 530 nm, emission at 590 nm) on a microplate reader (Synergy™ HT, BioTek). Values were graphed as % Control compared with media alone.

### Western Blotting

HUVEC cells were seeded into 6-well dishes and incubated at 37 °C in EGM-2 media with 2% FBS. When cells had reached 95–100% the media was exchanged and various concentrations of BN, BC194, or BC220 were added. For all samples (including 0 nM controls), extra DMSO was added to match the 0.05% (BN and BC194) and 0.2% (BC220) DMSO concentrations in the 1000 nM compound samples. For threonine rescue experiments, 2.5 to 10 mM threonine was also added to the cultures. Cells were replaced in 37 °C and incubated for 24 h. Cell monolayers were washed with PBS and harvested using CellLytic^TM^ M cell lysis reagent (Sigma) supplemented with complete Mini protease inhibitor tablets (Roche) according to the manufacturer’s instructions. For the thapsigargin control, the media was exchanged at the same time as for the borrelidin compounds, but 1 μg/ml thapsigargin (Sigma) was not added until 1 h before harvesting. Lysate protein concentrations were determined using Quick Start^TM^ Bradford Reagent (Bio-Rad) and stored at −80 °C until used. Lysates were resolved by 10% SDS-PAGE and transferred to nitrocellulose membranes for western blot analysis. Primary antibodies for phospo-eIF2α, cleaved-caspase 3, and β-tubulin were purchased from Cell Signaling and used at 1:1000. The secondary antibody, HRP-goat-anti-rabbit IgG (Santa Cruz), was used at 1:5000. Bands were detected by X-ray film or the VersaDoc 4000 MP (BioRad) using Pierce ECL blotting substrate or Supersignal West Femto reagent (Pierce) as needed. Densitometric values were determined using ImageJ lane analysis tools.

### *In vitro* Tube Formation Assay

Tube formation assays were performed as previously described^19^,^70^. Briefly HUVEC cells were cultured until 95–100% confluent and seeded in to 48-well plates coated with Matrigel^TM^ (BD Biosciences). Cells were incubated at 37 °C in EGM®-2 media with 2% FBS and various concentrations of BN, BC194, or BC220 for 4 to 8 h. For 0 nM (control), 0.1 nM, and 1 nM treated cultures, extra DMSO was added to match 0.01% (BN and BC194) or 0.05% (BC220) DMSO employed in the 10 nM samples. Cells were fixed with 10% neutral buffered formalin, stained with Oregon Green 488 phalloidin (Molecular Probes), and imaged via fluorescence microscopy. Tube structures were quantified using the Simple Neurite Tracer plug-in on ImageJ software (NIH).

### Chick Chorioallantoic Membrane Assay

CAM assays were performed as previously described^19^. Briefly, embryos from fertilized chicken eggs (Sunrise Farms, Catskill, NY) were cultured *ex-ova* in 10 cm^2^ tissue culture dishes at 37 °C until developmental day 10. Surgical sponges (Surgifoam) approximately 1 mm^3^ were then arranged within the outer third of the membrane avoiding major vessels. Compounds in 10 μl volumes were applied directly to the sponges at 24 h intervals over the course of 72 h. Images were taken daily using a Leica MZ6 stereomicroscope and scored using modified Intensity Scoring described previously^71^. Briefly, images of treated sponges were given a blinded score ranging from 0–5 corresponding to changes in vessel network density within the tissue surrounding the treated sponges. A score of 0 indicates no change in vessel density while 1–5 represent gradual increases in the number of vessels and directional changes towards the treatment. The total score for each experimental condition were averaged over at least 12 replicates.

### Cell Migration Assay

The donut cell migration assay was performed as previously described^49^. Briefly, polydimethylsiloxane (PDMS) donut-shaped gaskets were placed on fibronectin (BD Biosciences) -coated glass coverslips to form a 2 mm-diameter culture well into which 8 × 10^3^ HUVECs were seeded and allowed to adhere overnight. After removal of the gaskets, the circular HUVEC monolayers were washed twice with complete media to remove non-adherent cells, nuclei were labeled by incubation with Hoechst 33342 for 15 min, and the monolayers were re-fed with complete media containing vehicle (0.0001% DMSO) or BC194 at the indicated concentrations. Images of the circular monolayers were acquired after re-feeding and at the indicated times and image pairs were analyzed using a custom ImageJ macro to calculate the number of cells at each time point that have migrated beyond the original monolayer.

### Enzyme Preparation and Aminoacylation Experiments

Wildtype TARS, R442A TARS, and LARS were purified using procedures identical to those described previously^19^. R442A TARS was generated from wildtype human TARS by QuickChange II Site-Directed Mutagenesis (Qiagen) using the pET28a hctThrRS plasmid (obtained from Dieter Soll, Yale) with the forward primer 5′-CTGATTTTGGGGTTCACAGGACTCAC-3′ and its reverse complement. Activity data for wildtype TARS and LARS were previously reported^19^. R442A TARS aminoacylation kinetics were determined using methods outlined previously^19^ except that total protein concentration was used in place of the active site concentration owing to the protein’s inactivity.

The N-terminal His-tagged human TARS (residues 322–723) was constructed in a PET28a vector. The protein was expressed in strain BL21 (DE3) and induced with 0.2 mM IPTG for 20 h at 16 °C. The cell pellet (from 4–8 liters) was lysed in NTA-wash buffer (500 mM NaCl, 20 mM Tris-HCl pH 8.0, 30 mM imidazole), loaded onto a Ni-HiTrap column and washed with NTA-wash buffer. The protein was eluted with NTA-elution buffer (500 mM NaCl, 20 mM Tris-HCl pH 8.0, 250 mM imidazole). The eluted protein was further purified by a QAE anion exchange column with NaCl gradient. The peak fractions of the protein were then concentrated for crystallization.

### Crystallization and Structure Determination

Crystallization was done by the sitting drop method. To crystallize human TARS – BC194 complex, protein solution (10 mg/mL) was pre-mixed with 2 mM BC194 and 5 mM _L–_Thr at 4 °C. The protein was then crystallized by mixing 0.5 μL of protein solution with 0.5 μL of precipitant solution, containing 0.1 M Tris pH 8.0, and 8% PEG8000. After incubation at 18 °C for 3–7 days, the crystals were flash-frozen in liquid nitrogen for data collection with a cryo solution consisting of 0.08 M calcium acetate, 6.4% PEG4000, and 20% glycerol. Diffraction data were obtained from the Ls-cat 21-ID-G beamline at the Advanced Photon Source (APS, Argonne, IL).

A data set was integrated and scaled with HKL2000^72^. The structures were determined by molecular replacement based on the human TARS structure (pdb: 4P3N) in program Molrep^73^. After corrections for bulk solvent and overall B values, data were refined by iterative cycles of positional refinement and TLS refinement with PHENIX^74^ and model building with COOT^75^. All current models have good geometry and no residues are in the disallowed region of the Ramachandran plot. Data collection and model statistics are given in Table 1.

### *In Vitro* Translation Assay

The effects of BC194 and borrelidin on cell-free protein translation were assayed in a rabbit reticulocyte lysate (RRL) according to the manufacturer’s instructions (Promega), with the exceptions that no extra amino acid mix was added and 0.02 mg/ml firefly luciferase mRNA was used in the assays. Experiments were performed in triplicate and the data fit to the Gaddum/Schild EC_50_ shift equation.

### Zebrafish Handling and Treatments

This project utilized the transgenic lines Tg(flk1:eGFP) and Tg(flk1:dsRed) in which eGFP and dsRed expression respectively are controlled by the flk1 promoter. The Tg(flk1:eGFP) or Tg(flk1:dsRed) fish lines exhibit endothelial specific fluorescence starting 48 and 72 h after fertilization respectively. Due to its earlier expression the Tg(flk1:eGFP) line was used for all 24 hpe (~48 h after fertilization) fluorescence images; all other applications used the Tg(flk1:dsRed) line. Zebrafish were bred and maintained in accordance with approved University of Vermont IACUC standards for vertebrate animals and staged as previously described^76^. Fertilized embryos were transferred to glass dishes and allowed to develop at 25 °C until 8 to 12 somites in age. The fish were manually dechorionated and transferred to 24 well dishes containing 1 ml of egg water treated with 0.003% 1-phenyl 2-thiourea to inhibit pigment, and various concentrations of BN, BC194, BC220, or DMSO (vehicle control) and incubated at 28.5 °C for down stream analyses. For all conditions, extra DMSO was added where needed to match the 0.025% (BN and BC194) and 0.1% (BC220) DMSO concentrations present in the 5 μM compound samples. Dead and dying embryos were removed and the media exchanged for fresh egg water and compounds every 24 h.

For TARS morphant studies, a morpholino (5′-TAATTTAGCCACTAACCTGATCCCA-3′) was designed targeting the splice donor site within exon 3 of TARS mRNA. Blocking this site is predicted to join exon 2 to exon 4, introducing a frameshift and producing a stop codon five codons into exon 4. TARS morpholinos (1.5 mM) were injected into the yolk sac of Tg(flk1:eGFP) embryos at the one to four cell stage and incubated for 24 hours at 28.5 °C. The embryos were then dechorionated, transferred to fish egg water containing 0.003% PTU, and replaced at 28.5 °C. The successful knockdown of functional TARS was confirmed by RT-qPCR (described below) and RT-PCR using the following primers:

Exon 2, Forward: 5′-CAAGAATGCTGCTGGAGATG-3′

Exon 4, Reverse: 5′-CTTGTGCCTCTTCATCGTCA-3′

Imaging and aberrant vessel quantification were performed at 24 and 48 hpe as described below.

### Quantitative Assessment of Toxicity

Heart rate and body length data were acquired after the first 24 h of compound exposure using the Tg(flk1:dsRed) line and bright field microscopy. Heart rates were counted over 15 sec and converted to beats per min. For body length, images were taken using a Spot camera attached to a Nikon SMZ800 dissecting microscope at a 3X objective and analyzed with Spot v 5.1 software.

### Confocal Imaging

Embryos were live imaged with a 20x objective using step sizes of 2 μm after mounting in 0.5% low melt agarose on glass bottom dishes (Mat-Tek) on a Nikon Eclipse Ti inverted microscope. Stacks were subjected to a kalman stack filter in ImageJ and are presented as maximum intensity projections. Images were processed with Adobe Photoshop for brightness and contrast. For quantification of ISV ectopic branches, images were taken using an Olympus IX71 inverted fluorescence microscope. Abnormal branches and vessel defects associated with ISVs were counted within a region encompassing five ISVs anterior and posterior to the end of the yolk extension.

### Live-Imaging of Zebrafish Blood Flow

Zebrafish embryos of the Tg(flk:dsRed) line were treated for 48 hours with 0.025% DMSO or 5 μM BC194 as described above. Fluorescence images were acquired on a Nikon TE2000-PFS epifluorescence microscope through a 20X Plan-Fluor objective using wavelength-appropriate filters (Chroma Technologies) with a Clara CCD camera (Andor, Inc.). Acquisition was controlled using Nikon Elements software and images were adjusted for presentation using Elements or Fiji/ImageJ (http://imagej.nih.gov/ij/; W. Rasband, NIH).

### RNA Expression in Zebrafish

For compound treatments, embryos (~8 somites; Tg(flk:dsRed)) were manually dechorionated and incubated for 24 or 48 h in egg water containing varying concentrations of compounds at 28.5 °C. For morpholino validation, the anti-TARS morpholino was injected into the yolk sac at the one to four cell stage as described above. The fish were subsequently transferred in minimum volume to 1.5 mL microfuge tubes and then total RNA was obtained using Trizol:chloroform (Invitrogen). First strand cDNA was synthesized using Superscript II RT-PCR technology (Invitrogen) according to manufacturer’s instructions. RT-qPCR was performed using an ABI prism 7700 Sequence Detection System (Applied Biosystems) and TaqMan® Assay primer pairs (Applied Biosystems) for *tars*, *vegfaa*, *heyL*, *ephrinb2a*, and *act1a*. Relative mRNA expression was determined using a comparative CT (ΔΔCT) method with *act1a* as an endogenous control.

### Statistical Analysis

Unless noted otherwise, all experiments were repeated at least 3 times; n values are reported in the corresponding figure legend. Values are presented as means ± SEM. Data was analyzed by one- or two-way ANOVA and Tukey or Sidak test as indicated using GraphPad Prism v6 software. Significance was defined as P < 0.05.

## Additional Information

**Accession codes**: The atomic coordinates and structure factors of human TARS–BC194 has been deposited in the Protein Data Bank (PDB) with the accession codes 4TTV.

**How to cite this article**: Mirando, A. C. *et al.* Aminoacyl-tRNA synthetase dependent angiogenesis revealed by a bioengineered macrolide inhibitor. *Sci. Rep.* **5**, 13160; doi: 10.1038/srep13160 (2015).

## Supplementary Material

Supplementary Movie S1

Supplementary Movie S2

Supplementary Information

## Figures and Tables

**Table 1:** Crystallographic statistics of TARS–BC194 complex structures.

	Human ThrRS-BC194
Data collection	
Space group	*P2*_*1*_
Cell dimensions	
*a*, *b*, *c* (Å)	92.02, 134.61, 128.67
α, β, γ (°)	90.00, 90.10, 90.00
Resolution (Å)	50.00 – 2.80 (2.95 – 2.80)*
*R*_sym_ or *R*_merge_ (%)	14.7 (88.9)
*I*/s*I*	5.2 (1.3)
Completeness (%)	100.0 (100.0)
Redundancy	3.2 (3.2)
Refinement	
Resolution (Å)	50.00 – 2.80 (2.90 – 2.80)
No. reflections	76770 (7616)
*R*_work_/*R*_free_ (%)	25.1/28.7
No. atoms	
Protein	13045
Ligand	140
Solvent	62
*B*-factors (Å^2^)	
Protein	56.41
Ligand	49.18
Solvent	46.82
R.m.s. deviations	
Bond lengths (Å)	0.007
Bond angles (°)	1.149
Ramachandran plot	
*Most favored* [%]	96.4
*Additional allowed* [%]	3.6

^*^Values in parentheses are for highest-resolution shell.
