# Supplementary material for: Aminoacyl-tRNA synthetase dependent angiogenesis revealed by a bioengineered macrolide inhibitor
Source: Sci Rep. 2015 Aug 14;5:13160. doi: 10.1038/srep13160 (PMC4536658; doi:10.1038/srep13160)
Supplement: Supplementary Information [file srep13160-s3.pdf]

Supplementary Information for:

Aminoacyl-tRNA synthetase dependent angiogenesis revealed by a bioengineered macrolide inhibitor

Adam C. Mirando[1], Pengfei Fang[2], Tamara F. Williams[3], Linda C. Baldor[3], Alan K. Howe[3], Alicia M. Ebert[4], Barrie Wilkinson[6†], Karen M. Lounsbury[3], Min Guo[2], and Christopher S. Francklyn[1]*

[1]Department of Biochemistry, University of Vermont.

[2]Department of Cancer Biology, The Scripps Research Institute, Scripps Florida.

[3]Department of Pharmacology, University of Vermont.

[4]Department of Biology, University of Vermont

[5]Department of Pathology, University of Vermont

[6]Isomerase Therapeutics Ltd, Science Village, Chesterford Research Park, Cambridge CB10 1XL, UK

[†]Current address: John Innes Institute Centre, Norwich Research Park, Norwich NR4 7UH, UK

*Corresponding Author

email: Christopher.Francklyn@uvm.edu

Tel: 802-656-8450, Fax: 802-656-8220

Supplementary Table 1

Atoms_of_ Borrelidin_(BN)	Atoms_from _ThrRS	Distance (Å)	Interaction _Type	Conserved_ Interactions	Atoms_of_ BC194	Atoms_from_ThrRS	Distance (Å)	Interaction_Type
(BN)./C01[C]	411(MET)./CE[C]	4.28	Hydrophobic					
	540(TYR)./OH[O]	3.31	Van der waals	✓	(BC194)./C01[C]	540(TYR)./OH[O]	3.35	Van der waals
(BN)./OC1[O]	540(TYR)./CZ[C]	4.49	Van der waals					
	540(TYR)./OH[O]	3.13	H-bond	✓	(BC194)./OC1[O]	540(TYR)./OH[O]	3.44	H-bond
(BN)./C02[C]	411(MET)./CE[C]	4.33	Hydrophobic	✓	(BC194)./C02[C]	411(MET)./CE[C]	4.41	Hydrophobic
	540(TYR)./OH[O]	3.76	Van der waals	✓		540(TYR)./OH[O]	3.89	Van der waals
(BN)./C03[C]	540(TYR)./OH[O]	3.79	Van der waals	✓	(BC194)./C03[C]	540(TYR)./OH[O]	3.55	Van der waals
(BN)./OC3[O]	540(TYR)./CE1[C]	3.95	Van der waals	✓	(BC194)./OC3[O]	540(TYR)./CE2[C]	3.25	Van der waals
	540(TYR)./CZ[C]	3.71	Van der waals	✓		540(TYR)./CZ[C]	3.20	Van der waals
	540(TYR)./OH[O]	2.67	H-bond	✓		540(TYR)./OH[O]	2.33	H-bond
(BN)./CC4[C]	392(TYR)./CE2[C]	4.28	Hydrophobic	✓	(BC194)./CC4[C]	392(TYR)./CE1[C]	4.23	Hydrophobic
(BN)./C05[C]	392(TYR)./CD2[C]	4.13	Hydrophobic	✓	(BC194)./C05[C]	392(TYR)./CD1[C]	4.43	Hydrophobic
	392(TYR)./CE2[C]	4.23	Hydrophobic	✓		392(TYR)./CE1[C]	4.41	Hydrophobic
(BN)./C06[C]	540(TYR)./CE1[C]	4.18	Hydrophobic	✓	(BC194)./C06[C]	540(TYR)./CE2[C]	4.40	Hydrophobic
	540(TYR)./OH[O]	4.47	Van der waals		(BC194)./CC6[C]	391(HIS)./CB[C]	4.20	Hydrophobic
(BN)./CC6[C]	540(TYR)./CE1[C]	4.32	Hydrophobic			391(HIS)./CG[C]	4.45	Hydrophobic
	539(PHE)./CE2[C]	3.89	Hydrophobic	✓		539(PHE)./CE1[C]	4.07	Hydrophobic
(BN)./C07[C]	392(TYR)./CD2[C]	4.41	Hydrophobic	✓	(BC194)./C07[C]	392(TYR)./CD1[C]	4.40	Hydrophobic
	391(HIS)./CB[C]	4.23	Hydrophobic	✓		391(HIS)./CB[C]	4.22	Hydrophobic
(BN)./CC8[C]	391(HIS)./CB[C]	4.26	Hydrophobic		(BC194)./C08[C]	387(GLY)./O[O]	4.22	Van der waals
	391(HIS)./CD2[C]	4.48	Hydrophobic			391(HIS)./CB[C]	4.31	Hydrophobic
	387(GLY)./CA[C]	4.35	Hydrophobic	✓	(BC194)./CC8[C]	387(GLY)./CA[C]	4.40	Hydrophobic

	387(GLY)./C[C]	3.65	Hydrophobic	✓		387(GLY)./C[C]	3.74	Hydrophobic
	388(HIS)./N[N]	3.71	Van der waals	✓		388(HIS)./N[N]	3.93	Van der waals
	388(HIS)./CA[C]	3.86	Hydrophobic	✓		388(HIS)./CA[C]	4.00	Hydrophobic
	388(HIS)./CB[C]	4.49	Hydrophobic	✓		388(HIS)./CB[C]	4.45	Hydrophobic
	386(SER)./O[O]	4.26	Van der waals	✓		386(SER)./O[O]	4.06	Van der waals
	388(HIS)./CG[C]	3.89	Hydrophobic	✓		388(HIS)./CG[C]	3.68	Hydrophobic
	388(HIS)./ND1[N]	4.14	Van der waals	✓		388(HIS)./ND1[N]	3.66	Van der waals
	388(HIS)./CE1[C]	4.16	Hydrophobic	✓		388(HIS)./CE1[C]	3.60	Hydrophobic
	387(GLY)./O[O]	3.67	Van der waals	✓		387(GLY)./O[O]	3.66	Van der waals
	388(HIS)./CD2[C]	3.76	Hydrophobic	✓		388(HIS)./CD2[C]	3.61	Hydrophobic
	388(HIS)./NE2[N]	3.93	Van der waals	✓		388(HIS)./NE2[N]	3.56	Van der waals
(BN)./C09[C]	540(TYR)./CE1[C]	4.05	Hydrophobic	✓	(BC194)./C09[C]	540(TYR)./CE2[C]	4.46	Hydrophobic
	540(TYR)./CZ[C]	4.45	Hydrophobic			540(TYR)./CD2[C]	4.10	Hydrophobic
(BN)./C10[C]	386(SER)./O[O]	4.27	Van der waals	✓	(BC194)./C10[C]	386(SER)./O[O]	3.72	Van der waals
	540(TYR)./CE1[C]	4.08	Hydrophobic					
	540(TYR)./CD1[C]	4.24	Hydrophobic					
(BN)./CC10[C]	386(SER)./O[O]	4.20	Van der waals	✓	(BC194)./CC10[C]	386(SER)./O[O]	3.65	Van der waals
	540(TYR)./CE1[C]	3.83	Hydrophobic					
	540(TYR)./CG[C]	4.32	Hydrophobic					
	540(TYR)./CD1[C]	3.61	Hydrophobic	✓		540(TYR)./CD2[C]	4.32	Hydrophobic
	539(PHE)./CZ[C]	3.81	Hydrophobic	✓		539(PHE)./CZ[C]	4.06	Hydrophobic
(BN)./C11[C]	540(TYR)./CE2[C]	4.05	Hydrophobic	✓	(BC194)./C11[C]	540(TYR)./CE2[C]	4.36	Hydrophobic
	540(TYR)./CE1[C]	3.78	Hydrophobic					
	540(TYR)./CZ[C]	3.85	Hydrophobic					
	540(TYR)./OH[O]	4.40	Van der waals					

	540(TYR)./CG[C]	4.14	Hydrophobic	✓		540(TYR)./CG[C]	4.40	Hydrophobic
	540(TYR)./CD1[C]	3.93	Hydrophobic					
	540(TYR)./CD2[C]	4.18	Hydrophobic	✓		540(TYR)./CD2[C]	4.30	Hydrophobic
	564(ASP)./CG[C]	4.31	Hydrophobic	✓		564(ASP)./CG[C]	4.46	Hydrophobic
	564(ASP)./OD2[O]	3.26	Van der waals	✓		564(ASP)./OD2[O]	3.45	Van der waals
(BN)./OC11[O]	567(LEU)./CG[C]	4.21	Van der waals	✓	(BC194)./OC11[O]	567(LEU)./CG[C]	4.15	Van der waals
	567(LEU)./CD1[C]	3.74	Van der waals	✓		567(LEU)./CD1[C]	3.52	Van der waals
	567(LEU)./CD2[C]	4.04	Van der waals	✓		567(LEU)./CD2[C]	4.30	Van der waals
	567(LEU)./CB[C]	4.23	Van der waals	✓		567(LEU)./CB[C]	4.04	Van der waals
	540(TYR)./CG[C]	4.19	Van der waals			567(LEU)./N[N]	4.24	Van der waals
	540(TYR)./CD1[C]	4.34	Van der waals			567(LEU)./CA[C]	4.46	Van der waals
	540(TYR)./CD2[C]	4.29	Van der waals			386(SER)./O[O]	4.14	Van der waals
	564(ASP)./CG[C]	3.38	Van der waals	✓		564(ASP)./CG[C]	3.81	Van der waals
	564(ASP)./OD1[O]	3.63	Van der waals	✓		564(ASP)./OD1[O]	3.87	Van der waals
	564(ASP)./OD2[O]	2.42	H-bond	✓		564(ASP)./OD2[O]	2.97	H-bond
(BN)./C12[C]	567(LEU)./CD2[C]	4.41	Hydrophobic	✓	(BC194)./C12[C]	567(LEU)./CD2[C]	4.50	Hydrophobic
	590(HIS)./CE1[C]	3.97	Hydrophobic	✓		590(HIS)./CE1[C]	4.36	Hydrophobic
	590(HIS)./NE2[N]	3.98	Van der waals					
	540(TYR)./CZ[C]	4.39	Hydrophobic					
	564(ASP)./OD2[O]	3.59	Van der waals	✓		564(ASP)./OD2[O]	3.55	Van der waals
(BN)./CC12[C]	388(HIS)./CE1[C]	4.24	Hydrophobic	✓	(BC194)./CC12[C]	388(HIS)./CE1[C]	4.24	Hydrophobic
	388(HIS)./NE2[N]	4.48	Van der					

			waals					
	413(CYS)./CB[C]	4.20	Hydrophobic	✓		413(CYS)./CB[C]	4.37	Hydrophobic
	413(CYS)./SG[S]	4.07	Van der waals	✓		413(CYS)./SG[S]	4.41	Van der waals
	567(LEU)./CD1[C]	4.26	Hydrophobic	✓		567(LEU)./CD1[C]	3.93	Hydrophobic
	567(LEU)./CD2[C]	3.83	Hydrophobic	✓		567(LEU)./CD2[C]	3.82	Hydrophobic
	590(HIS)./CE1[C]	4.08	Hydrophobic	✓		590(HIS)./CE1[C]	4.28	Hydrophobic
	590(HIS)./NE2[N]	4.44	Van der waals					
	411(MET)./SD[S]	4.44	Van der waals			567(LEU)./CG[C]	4.46	Hydrophobic
	564(ASP)./OD2[O]	4.13	Van der waals	✓		564(ASP)./OD2[O]	4.10	Van der waals
(BN)./NC12[N]	388(HIS)./CE1[C]	3.49	Van der waals	✓	(BC194)./NC12[N]	388(HIS)./CE1[C]	3.70	Van der waals
	388(HIS)./NE2[N]	4.00	Van der waals			414(PRO)./CG[C]	4.46	Van der waals
	388(HIS)./ND1[N]	4.49	Van der waals			414(PRO)./CD[C]	4.47	Van der waals
	413(CYS)./CB[C]	3.60	Van der waals	✓		413(CYS)./CB[C]	3.64	Van der waals
	413(CYS)./SG[S]	3.84	Van der waals	✓		413(CYS)./SG[S]	4.03	Van der waals
	567(LEU)./CG[C]	4.39	Van der waals	✓		567(LEU)./CG[C]	4.15	Van der waals
	567(LEU)./CD1[C]	3.87	Van der waals	✓		567(LEU)./CD1[C]	3.57	Van der waals
	567(LEU)./CD2[C]	3.60	Van der waals	✓		567(LEU)./CD2[C]	3.47	Van der waals
	411(MET)./SD[S]	4.50	Van der waals					
(BN)./C13[C]	540(TYR)./CZ[C]	3.76	Hydrophobic	✓	(BC194)./C13[C]	540(TYR)./CZ[C]	4.03	Hydrophobic
	540(TYR)./CE1[C]	4.40	Hydrophobic	✓		540(TYR)./CE1[C]	4.14	Hydrophobic
	540(TYR)./OH[O]	3.64	Van der	✓		540(TYR)./OH[O]	4.12	Van der waals

			waals					
	540(TYR)./CE2[C]	3.95	Hydrophobic					
	564(ASP)./OD2[O]	3.91	Van der waals	✓		564(ASP)./OD2[O]	3.70	Van der waals
	590(HIS)./CE1[C]	3.64	Hydrophobic	✓		590(HIS)./CE1[C]	4.02	Hydrophobic
	590(HIS)./NE2[N]	3.53	Van der waals	✓		590(HIS)./NE2[N]	4.10	Van der waals
	590(HIS)./CD2[C]	4.17	Hydrophobic					
	590(HIS)./ND1[N]	4.29	Van der waals					
(BN)./C14[C]	413(CYS)./SG[S]	3.96	Van der waals	✓	(BC194)./C14[C]	413(CYS)./SG[S]	4.02	Van der waals
	590(HIS)./CG[C]	3.87	Hydrophobic	✓		590(HIS)./CG[C]	4.33	Hydrophobic
	590(HIS)./CD2[C]	3.83	Hydrophobic	✓		590(HIS)./CD2[C]	4.35	Hydrophobic
	590(HIS)./ND1[N]	3.57	Van der waals	✓		590(HIS)./ND1[N]	3.78	Van der waals
	590(HIS)./CE1[C]	3.37	Hydrophobic	✓		590(HIS)./CE1[C]	3.47	Hydrophobic
	590(HIS)./NE2[N]	3.52	Van der waals	✓		590(HIS)./NE2[N]	3.84	Van der waals
	540(TYR)./OH[O]	4.04	Van der waals	✓		540(TYR)./OH[O]	4.37	Van der waals
	411(MET)./SD[S]	4.18	Van der waals	✓		411(MET)./SD[S]	4.00	Van der waals
	411(MET)./CE[C]	3.65	Hydrophobic			564(ASP)./OD2[O]	4.46	Van der waals
	(ZN)./ZN[ZN]	4.00	Van der waals					
(BN)./C15[C]	540(TYR)./OH[O]	3.27	Van der waals	✓	(BC194)./C15[C]	540(TYR)./OH[O]	3.45	Van der waals
	540(TYR)./CE2[C]	4.17	Hydrophobic	✓		540(TYR)./CZ[C]	3.98	Hydrophobic
	540(TYR)./CZ[C]	4.02	Hydrophobic	✓		540(TYR)./CE1[C]	4.08	Hydrophobic
	590(HIS)./CG[C]	3.80	Hydrophobic	✓		590(HIS)./CG[C]	4.15	Hydrophobic
	590(HIS)./CD2[C]	3.71	Hydrophobic	✓		590(HIS)./CD2[C]	4.18	Hydrophobic
	590(HIS)./ND1[N]	3.96	Van der waals	✓		590(HIS)./ND1[N]	4.00	Van der waals

	590(HIS)./CE1[C]	3.99	Hydrophobic	✓		590(HIS)./CE1[C]	3.97	Hydrophobic
	590(HIS)./NE2[N]	3.83	Van der waals	✓		590(HIS)./NE2[N]	4.07	Van der waals
	590(HIS)./CB[C]	4.43	Hydrophobic					
	411(MET)./CE[C]	4.01	Hydrophobic					
	562(GLN)./CG[C]	4.38	Hydrophobic					
(BN)./C16[C]	590(HIS)./CB[C]	3.87	Hydrophobic	✓	(BC194)./C16[C]	590(HIS)./CB[C]	4.09	Hydrophobic
	590(HIS)./CG[C]	3.78	Hydrophobic	✓		590(HIS)./CG[C]	3.85	Hydrophobic
	590(HIS)./CD2[C]	4.06	Hydrophobic	✓		590(HIS)./CD2[C]	4.28	Hydrophobic
	590(HIS)./ND1[N]	4.16	Van der waals	✓		590(HIS)./ND1[N]	3.87	Van der waals
	562(GLN)./CG[C]	4.32	Hydrophobic			590(HIS)./CE1[C]	4.32	Hydrophobic
	(ZN)./ZN[ZN]	4.31	Van der waals	✓		(ZN)./ZN[Zn]	4.27	Van der waals
	540(TYR)./OH[O]	4.14	Van der waals	✓		540(TYR)./OH[O]	4.15	Van der waals
	411(MET)./CE[C]	4.06	Hydrophobic					
(BN)./C17[C]	540(TYR)./OH[O]	4.21	Van der waals	✓	(BC194)./C17[C]	540(TYR)./OH[O]	3.99	Van der waals
	411(MET)./CE[C]	3.96	Hydrophobic					
(BN)./O18[O]	411(MET)./CE[C]	3.23	Van der waals	✓	(BC194)./O18[O]	411(MET)./CE[C]	3.79	Van der waals
	540(TYR)./OH[O]	3.90	Van der waals	✓		540(TYR)./OH[O]	3.56	Van der waals
(BN)./C18[C]	411(MET)./CE[C]	3.70	Hydrophobic	✓	(BC194)./C18[C]	411(MET)./CE[C]	4.43	Hydrophobic
(BN)./C19[C]	590(HIS)./CB[C]	3.99	Hydrophobic	✓	(BC194)./C19[C]	590(HIS)./CB[C]	4.25	Hydrophobic
	560(THR)./CG2[C]	3.91	Hydrophobic	✓		560(THR)./CG2[C]	3.95	Hydrophobic
	(ZN)./ZN[ZN]	4.48	Van der waals					
(BN)./C20[C]	592(ALA)./CB[C]	4.34	Hydrophobic					
	460(GLN)./NE2[N]	3.83	Van der waals					
	560(THR)./CG2[C]	3.07	Hydrophobic					

(BN)./C21[C]	560(THR)./CG2[C]	3.74	Hydrophobic	✓	(BC194)./C21[C]	560(THR)./CG2[C]	3.47	Hydrophobic
	460(GLN)./NE2[N]	3.29	Van der waals					
(BN)./C22[C]	460(GLN)./NE2[N]	4.09	Van der waals					
	411(MET)./CE[C]	4.38	Hydrophobic					
(BN)./C23[C]	460(GLN)./NE2[N]	3.70	Van der waals	✓	(BC194)./C23[C]	460(GLN)./NE2[N]	4.48	Van der waals
	442(ARG)./CZ[C]	4.08	Hydrophobic	✓		442(ARG)./CZ[C]	4.33	Hydrophobic
	442(ARG)./NH1[N]	3.76	Van der waals	✓		442(ARG)./NH1[N]	4.16	Van der waals
	411(MET)./CE[C]	3.77	Hydrophobic	✓		411(MET)./CE[C]	4.30	Hydrophobic
	442(ARG)./NH2[N]	3.45	Van der waals	✓		442(ARG)./NH2[N]	3.56	Van der waals
(BN)./OC23[O]	460(GLN)./CD[C]	4.04	Van der waals	✓	(BC194)./OC23[O]	460(GLN)./CD[C]	4.48	Van der waals
	460(GLN)./NE2[N]	3.02	H-bond	✓		460(GLN)./NE2[N]	3.70	Van der waals
	460(GLN)./OE1[O]	4.27	Van der waals	✓		460(GLN)./OE1[O]	4.39	Van der waals
	442(ARG)./CZ[C]	3.52	Van der waals	✓		442(ARG)./CZ[C]	3.90	Van der waals
	442(ARG)./NH1[N]	3.60	Van der waals	✓		442(ARG)./NH1[N]	4.06	Van der waals
	411(MET)./CE[C]	3.27	Van der waals	✓		411(MET)./CE[C]	4.10	Van der waals
	442(ARG)./NH2[N]	2.59	H-bond	✓		442(ARG)./NH2[N]	2.88	H-bond
(BN)./OT[O]	442(ARG)./CZ[C]	3.83	Van der waals	✓	(BC194)./OT[O]	442(ARG)./CZ[C]	3.93	Van der waals
	442(ARG)./NH1[N]	3.15	H-bond	✓		442(ARG)./NH1[N]	3.44	H-bond
	411(MET)./CE[C]	4.34	Van der waals					
	442(ARG)./NH2[N]	3.60	Van der waals	✓		442(ARG)./NH2[N]	3.54	Van der waals

**Total BN-ThrRS interactions**	**155**
**Total BC194-ThrRS interactions**	**123**
**Total shared interactions**	**110**

**Note:**	
H-bond	between an "O" and another "O"/"N", within 2.2-3.5 Å
Hydrophobic	between 2 "C" atoms, within 4.5 Å
Van der waals	any other interactions within 4.5 Å

Supplementary Figure Legends

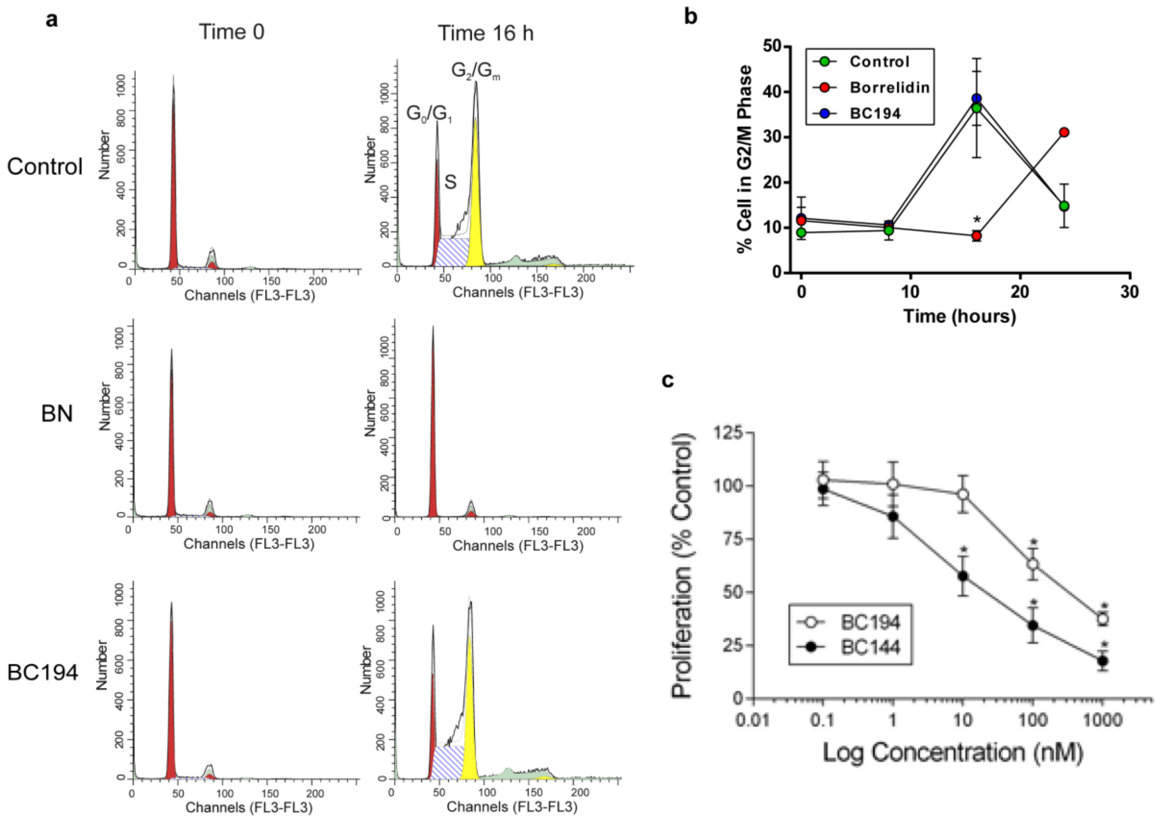

Supplementary Figure S1│ Borrelidin elicits toxic responses related to cell cycle progression, amino acid sensing, and apoptosis in endothelial cells at lower concentrations than BC194. (**a**) Cell-cycle analysis of synchronized HUVECs treated with serum containing BN or BC194 using flow cytometry. (**b**) Percentage of cells in G2/M phase following treatment with BN or BC194. HUVEC cells were treated with 10 nM BN or BC194, harvested at 0, 8, 16, and 24 h, and stained with propidium iodide. The cells in G2/M were determined by gating using the 16h serum control; mean ± SEM, n=3, *p<0.05 (one-way ANOVA, Tukey Test). (**c**) The effects of BN and BC194 on HUVEC proliferation. Cells were exposed to the indicate concentrations of compounds for 48 h and the relative proliferation measured using an alamarBlue® assay.

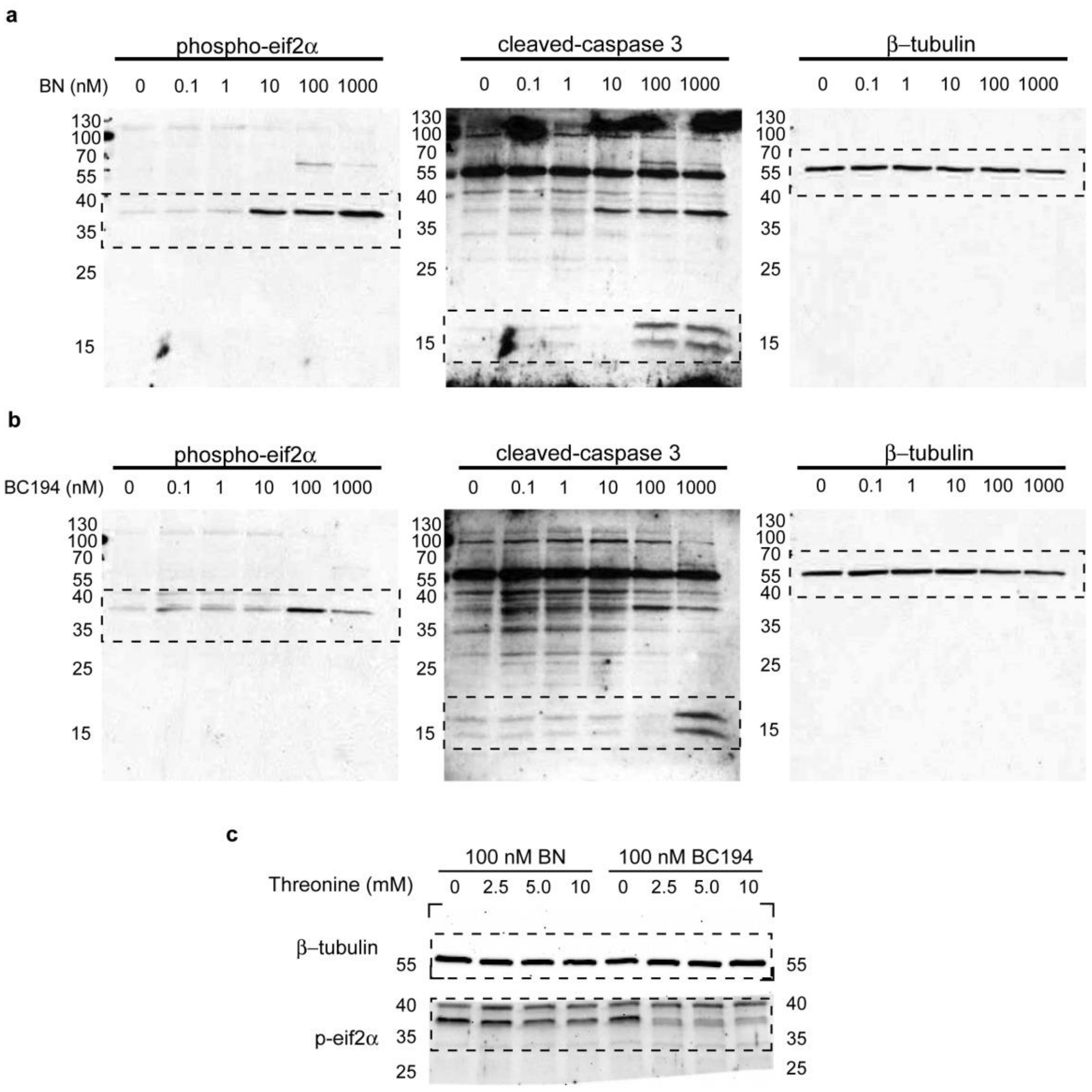

Supplementary Figure S2│ The cytotoxicity of borrelidin is linked to the induction of the amino acid starvation response. (**a, b**) Full size blots of cropped images used in to investigate the induction of amino acid starvation and apoptosis in HUVECs following treatment with BN (**a**) and BC194 (**b**) (See **Figures 3a** and **3d**). To obtain each image, the same blot membrane was probed sequentially with primary antibodies against phospho-eIF2α, β-tubulin, and cleaved-caspase 3, in that order. Note that final blot for caspase 3 contains the signals for phospho-eIF2α and β-tubulin, which were detected earlier. Cropped regions are designated by dotted lines. (**c**) Full size blot for cropped images used in the threonine rescue experiment (**Figure 3g**). The blot membrane was cut horizontally into three sections at the 25, 40, and 70 kDa MW markers and then resulting membrane sections probed individually with the indicated primary antibodies. Cropped regions are designated by dotted lines. The membrane section derived from the region of the acryamide gel corresponding to proteins migrating with a molecular weight less than 25 kD was not used in the analysis, and is not included in the above figure.

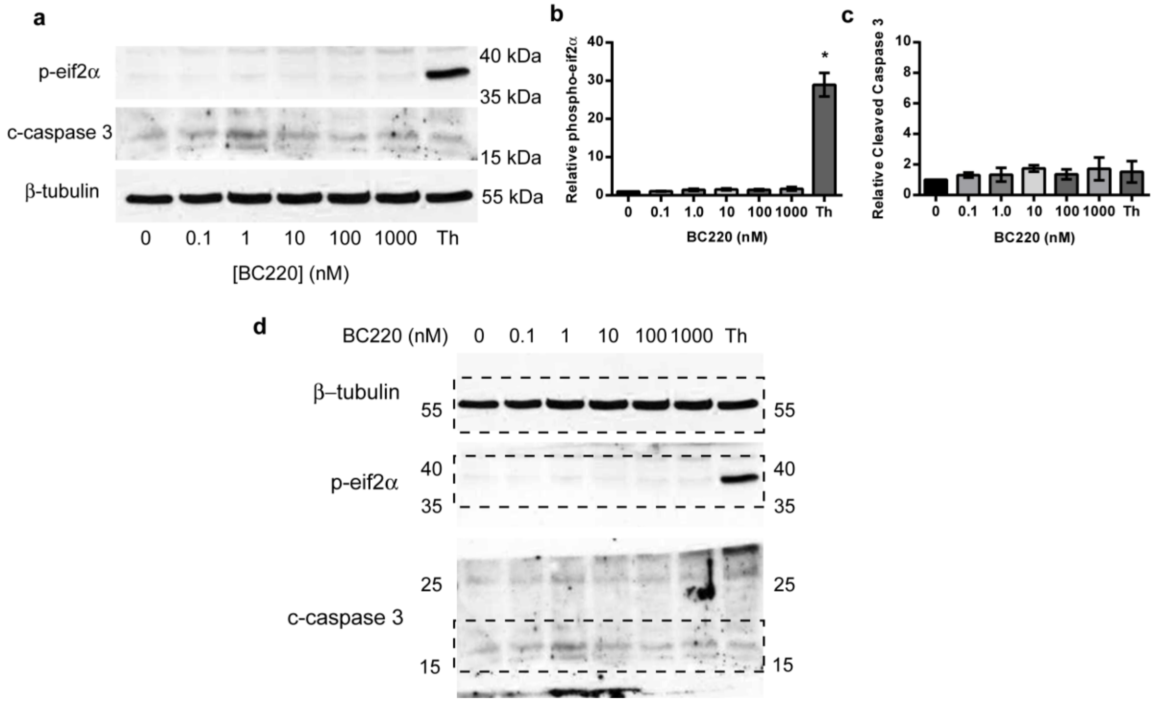

Supplementary Figure S3│ BC220 does not induce amino acid starvation responses. (**a-d**) Western blots of HUVEC extracts exposed to BC220 at the indicated concentrations using antibodies against phospho-eif2α and cleaved-caspase 3. Cultures were standardized to a concentration of 0.2% DMSO. (**a**). Thapsigargin (Th; 1 µg/ml) was used as a positive control for amino acid starvation. Images were cropped from the same blot membrane as indicated in **d**. Protein values for phospho-eIF2α (**b**) and cleaved-caspase 3 (**c**) were quantified relative to the β-tubulin loading control; mean ± SEM, n≥3, *$p<0.0001$ relative to 0 nM (one-way ANOVA, Tukey Test). (**d**) Full size blots for western blots shown in **a**. The blot membrane was cut horizontally into thirds at the 25, 40, and 70 kDa MW markers, and then each section was probed individually with the primary antibody indicated at the left. Dotted lines indicate cropped regions.

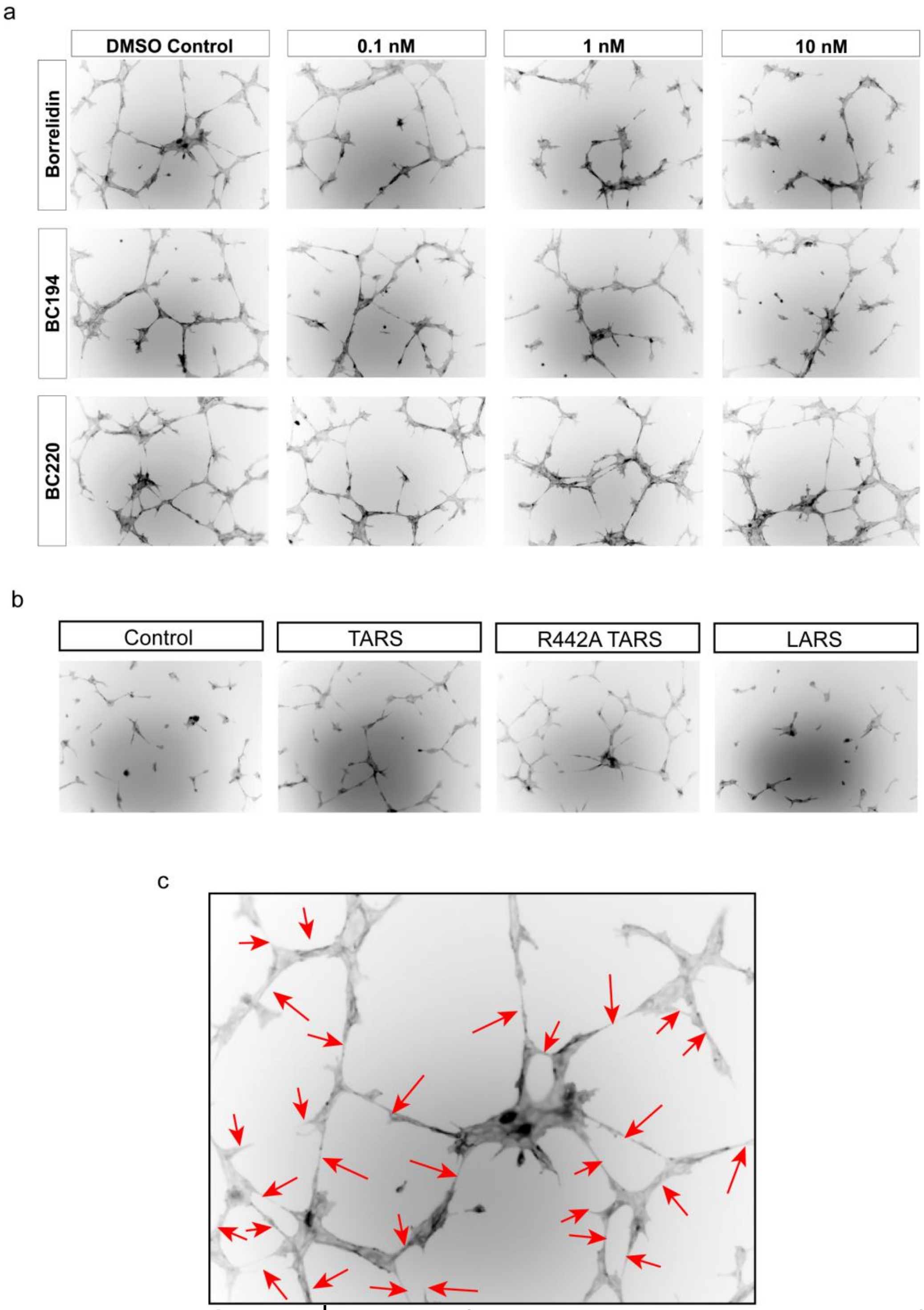

Supplementary Figure S4│ BN and BC194 exhibit comparable inhibition of angiogenesis at subtoxic levels. (**a, b**) Representative images of HUVEC branches that were quantitated to construct Figure 4a (**a**) and Figure 4d (**b**). HUVECs were plated on matrigel in full EGM-2 (2% FBS) media and exposed to the indicated concentrations of BN, BC194, and BC220 for 4-6 h. (**c**) A representative tube formation image identifying structures that were scored (Red arrows) as positive branches for the quantitations.

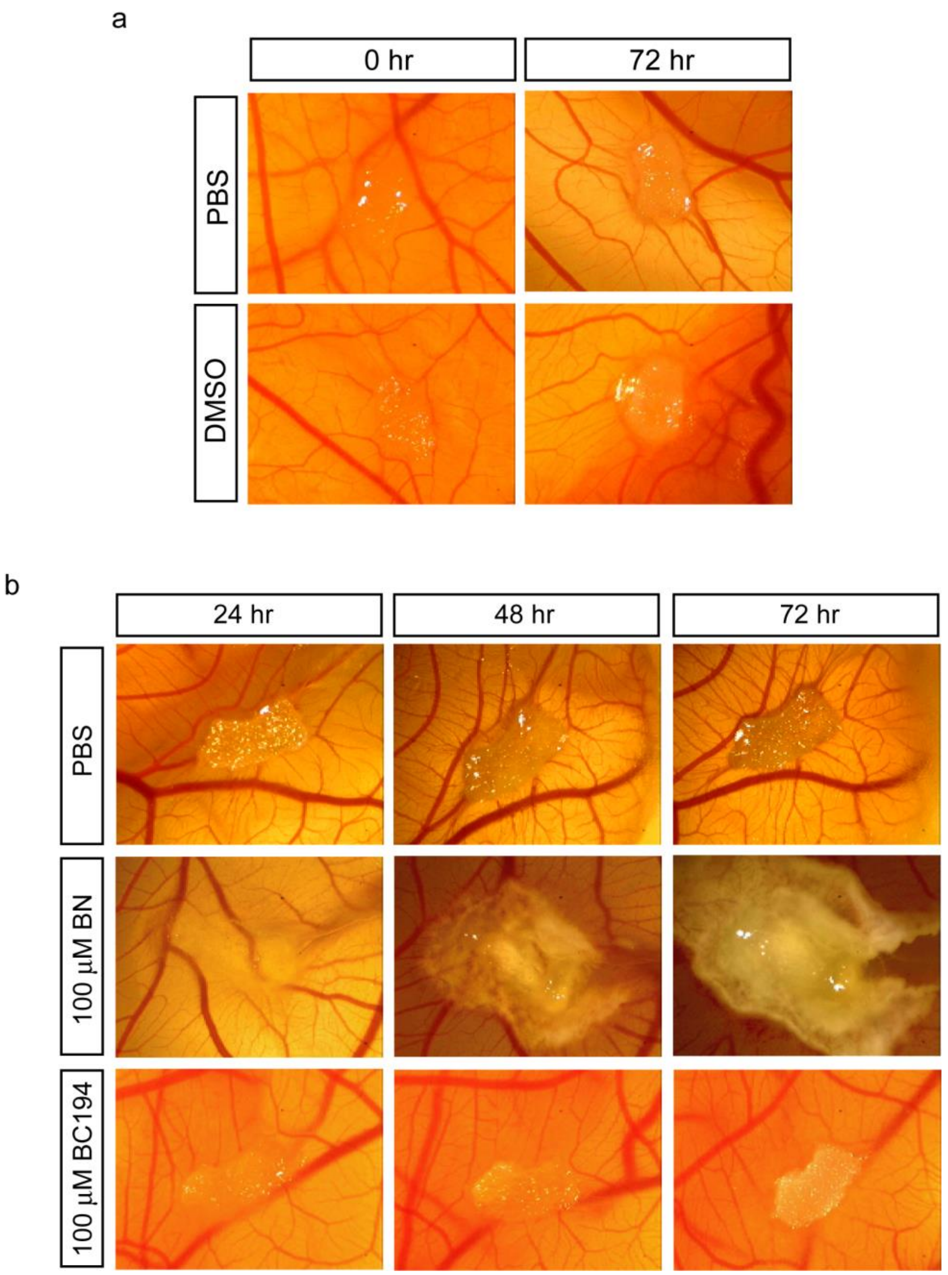

Supplementary Figure S5│ BN and BC194 exhibit comparable inhibition of angiogenesis at subtoxic levels. (**a**) Representative CAM images comparing DMSO (0.01%) and PBS treatments at 0 and 72 hrs. (**b**) Representative images of CAM comparing PBS, BN, and BC194 at 100 μM after exposure for 24, 48, and 72 hrs.

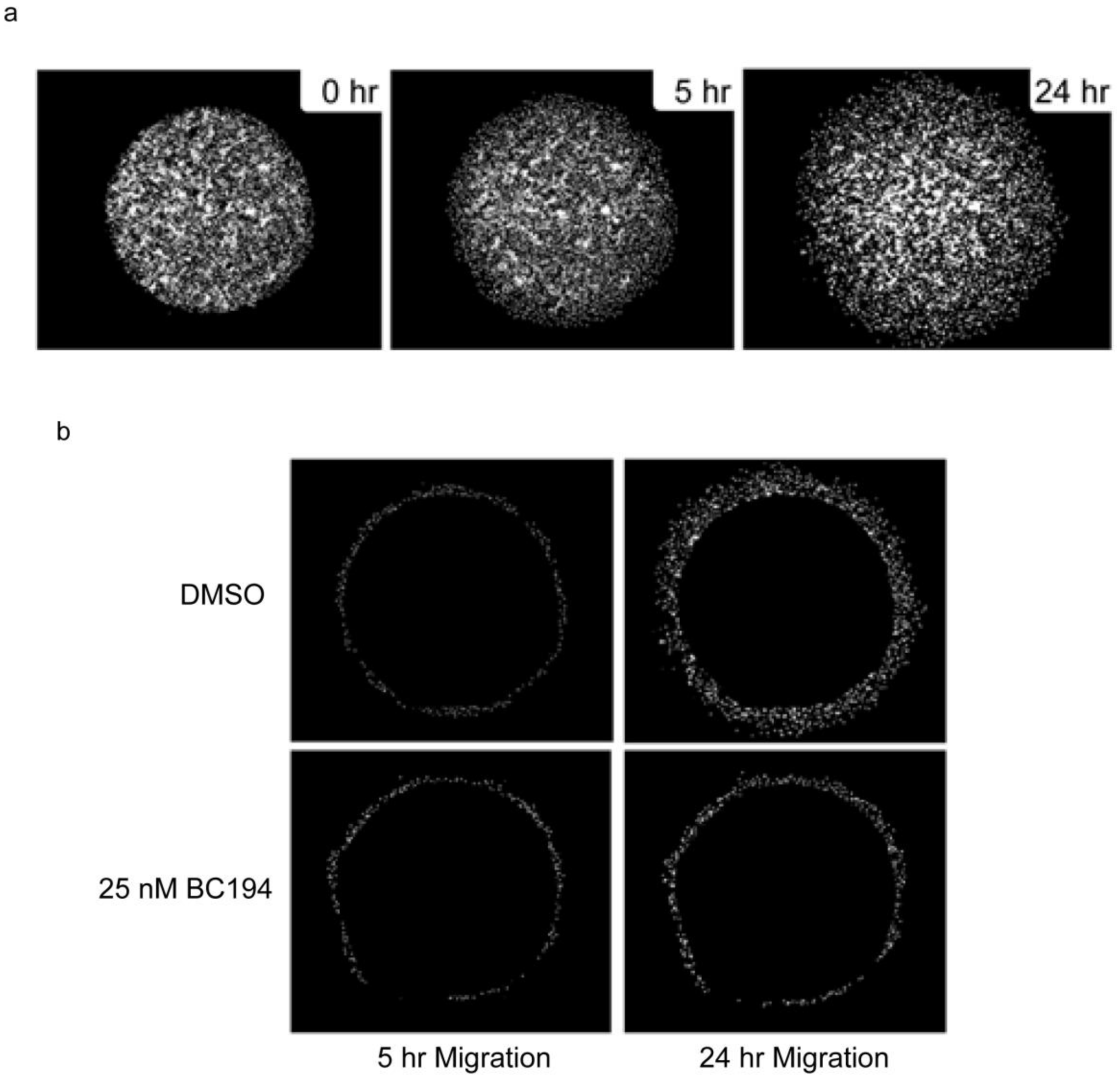

Supplementary Figure S6│ BN and BC194 exhibit comparable inhibition of angiogenesis at subtoxic levels. (**a, b**) Representative images of HUVEC cells in the donut migration assay. (**a**) Images of Hoechst 33342-labeled HUVEC cells (DMSO-treated) 0, 5, and 24 h after removal of PDMS gasket to demonstrate cell migration technique. (**b**) Images of cells treated with DMSO (upper two) or 25 nM BC194 (lower two) outside the original PDMS gasket boundary after 5 and 24 h using cell subtraction.

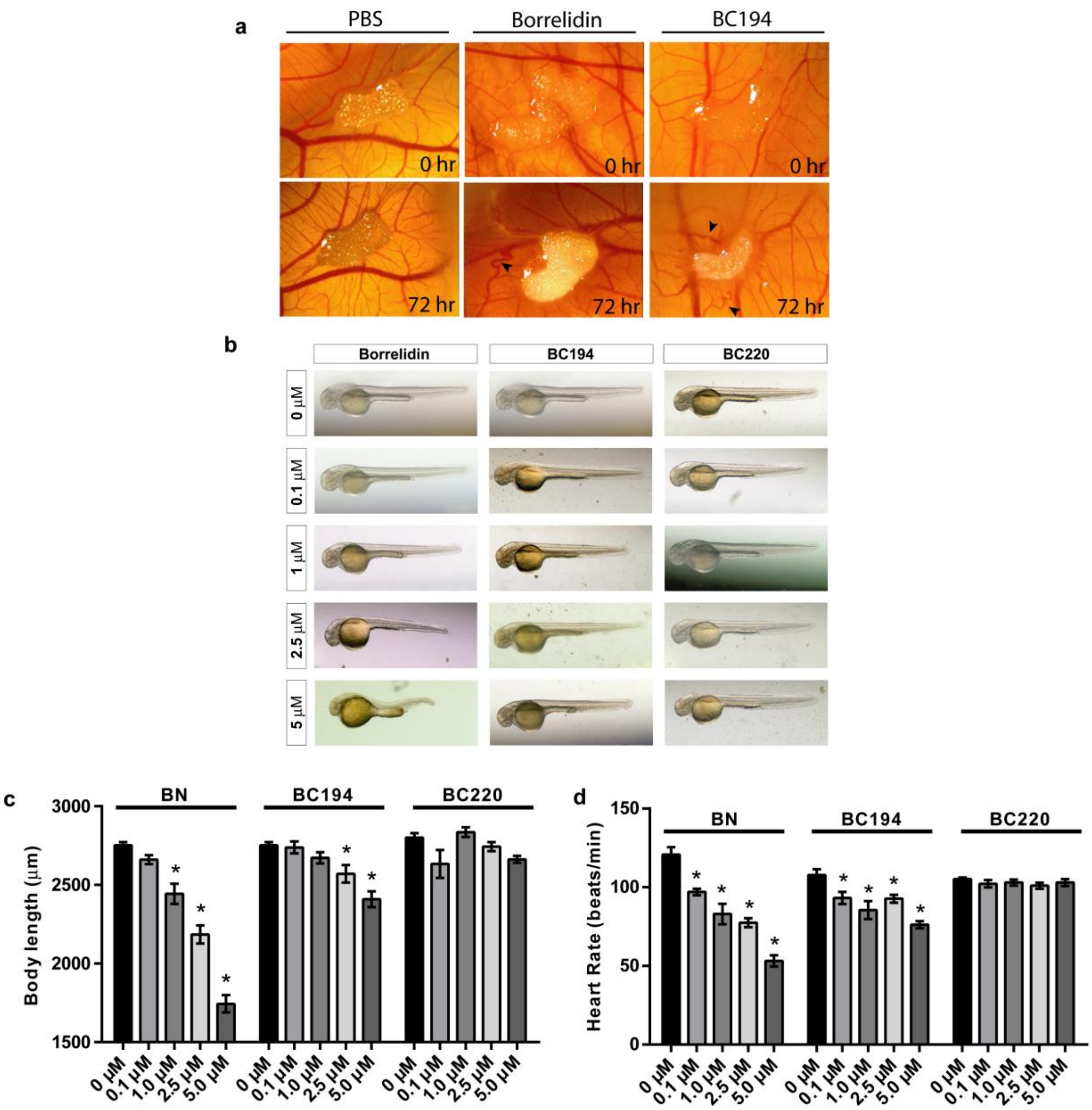

Supplementary Figure S7│ Exposure to BN and BC194 results in vascular defects and mis-patterning. (**a**) Representative CAM images showing rapid changes in vessel directionality, indicated by black arrows, before and after 72 hour treatment with PBS control, 1 µM BN, or 1 µM BC194. (**b**) Whole body images of 24 hpe zebrafish embryos treated with various concentrations of BN, BC194, and BC220 for 24 h. Extra DMSO was added to all samples, including 0 µM, as required to match the highest concentration used for each compound (0.025% for BN and BC194 and 0.1% in BC220). (**c, d**) Histograms showing the effects of BN, BC194, and BC220 exposure on zebrafish body length (**c**) and heart rate (**d**). Dechorionated embryos were incubated for 24 hours in egg water containing the indicated compounds. Body length measurements were obtained by analysis of bright-field images using Spot v 5.1 software; mean ± SEM, n=11, *p<0.05 relative to 0 nM (one-way ANOVA, Tukey Test). Heart rates were determined using bright-field microscopy to count heart beats over 15 second intervals; mean ± SEM, n=16, *p<0.05 relative to 0 nM (one-way ANOVA, Tukey Test).

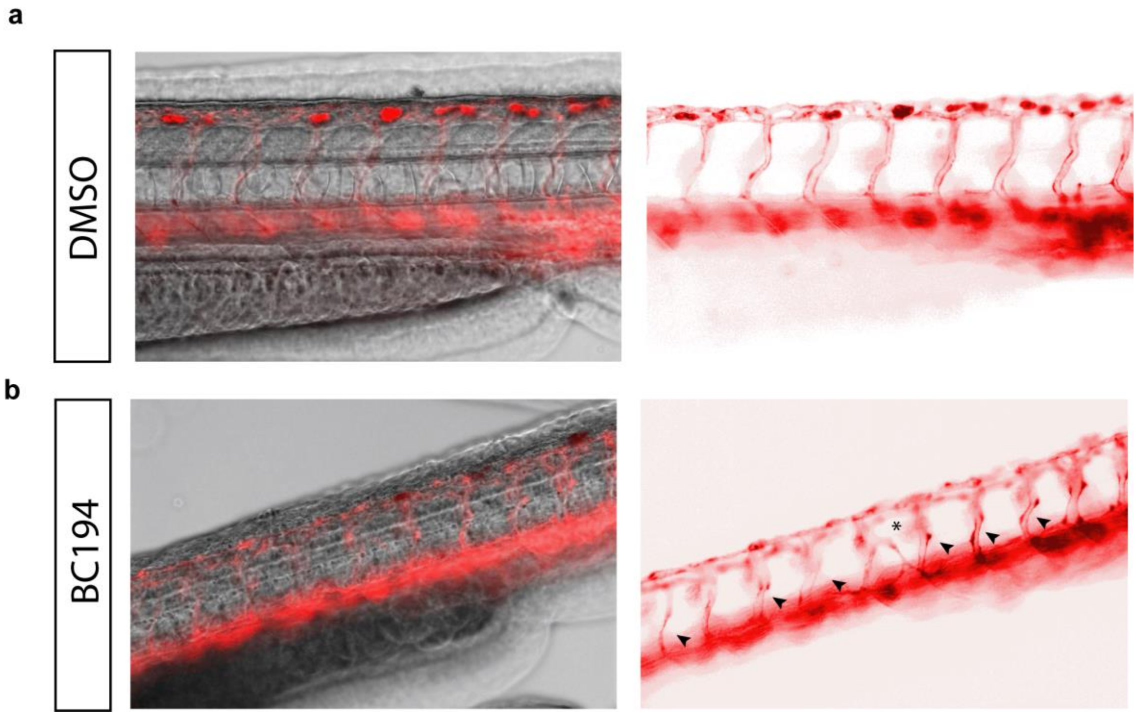

Supplementary Figure S8│ Treatment with BC194 inhibits blood-flow through zebrafish ISVs. (**a**, **b**) Still frame image from videos of trunk blood flow in zebrafish embryos 72 hours after fertilization (left) and fluorescence image of blood vessels (right) treated with DMSO (**a**) or 5 µM BC194 (**b**) for 48 h. Non-functioning lumens and ectopic branches are designated with black arrows and asterisks respectively.

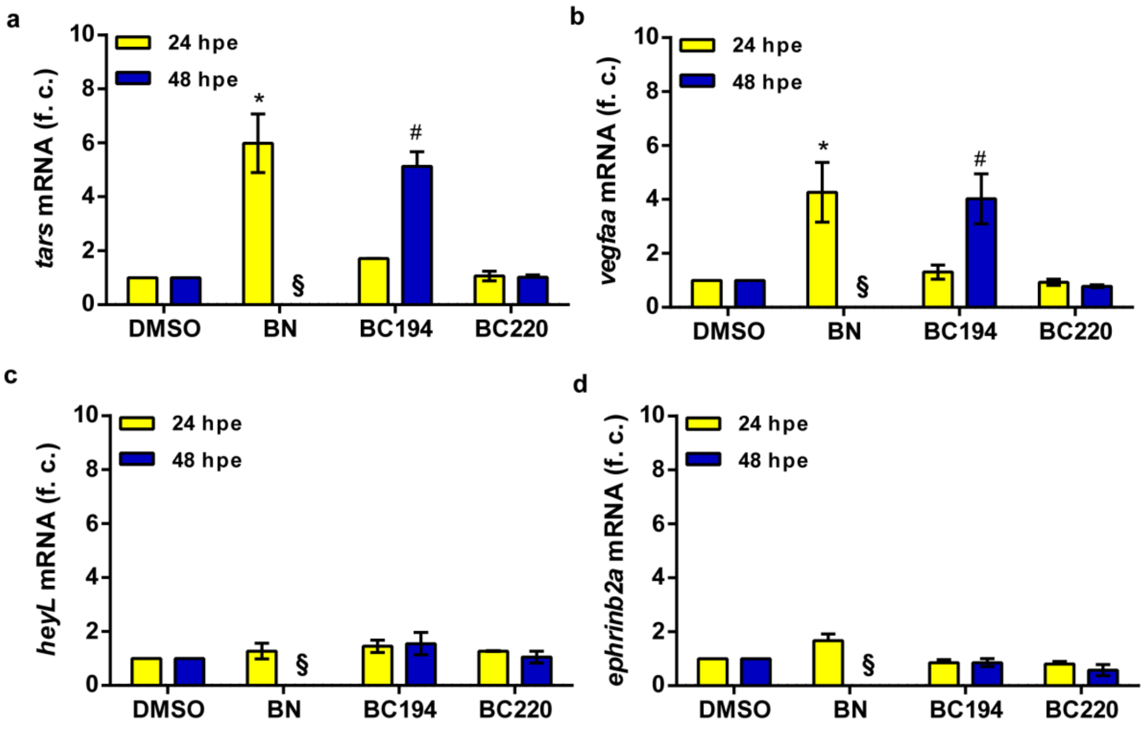

Supplementary Figure S9│ BN and BC194 treatment affects gene expression. (**a-d**) RT-qPCR values for the expression of *tars* (**a**), *vegfaa* (**b**), *ephrinb2a* (**c**), and heyL (**d**) at 24 and 48 hpe. Dechorionated embryos (15 somites) were incubated in egg water containing the indicated compounds. Total RNA was extracted by trizol/chloroform and the expression levels relative to *actb were* determined using the ΔΔCT method; mean ± SEM, n=3, *#p<0.05 relative to DMSO at 24 and 48 hpe respectively (one-way ANOVA, Tukey Test). § denotes that BN-treated embryos rarely survived to 48 hpe and were not included in this figure.

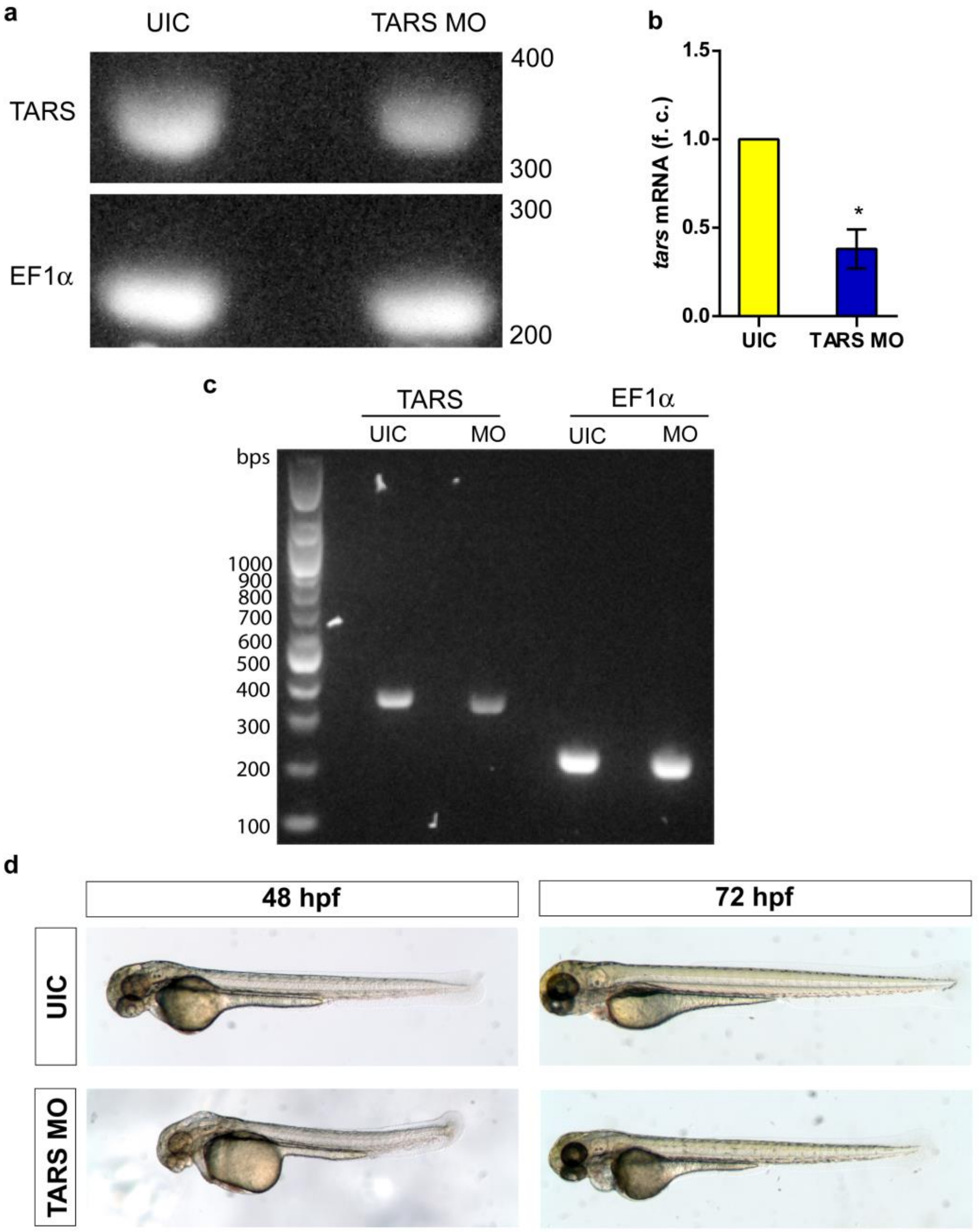

Supplementary Figure S10│Depletion of TARS elicits a similar vascular phenotype to BN and BC194 inhibition. (**a-b**) RT-PCR (**a**) and RT-qPCR (**b**) of trizol/chloroform extracted mRNA from uninjected controls (UIC) and TARS morphants (MO) validate the reduction of TARS levels by morpholino injection. (**a**) Whole fish mRNA (48 hpe) was amplified by RT-PCR with gene specific primers. The TARS (338 bp) and EF1α control amplicons were run on 1.5% agarose to show differences in relative intensities between uninjected controls (UIC) and morphant fish. (**b**) cDNA was generated from isolated mRNA using oligo-dT primers and the tars expression relative to EF1α was determined by the ΔΔCT method; mean ± SEM, n=2, *p<0.05 relative to UIC (unpaired t-test). (**c**) Full size gel for image shown in **a**. (**d**) Whole body images of UIC and TARS morphant fish at 24 and 48 hpe.

Supplementary Movie S1│Live imaging of zebrafish blood-flow following treatment with DMSO. Tg(Flk1:dsRed) embryos (15 somites) were dechorionated and treated for 48 hours with 0.025% DMSO. Videos were taken by bright-field microscopy with a 20x pan-fluor objective. Video frames are composites of a single fluorescence image superimposed over the bright-field images. See also Figure S4, part a.

Supplementary Movie S2│Live imaging of zebrafish blood-flow following treatment with BC194. Tg(Flk1:dsRed) embryos (15 somites) were dechorionated and treated for 48 hours with 5 µM BC194 in 0.025% DMSO. Videos were taken by bright-field microscopy with a 20x pan-fluor objective. Video frames are composites of a single fluorescence image superimposed over the bright-field images. See also Figure S4, part b.
